# MITF Is Regulated by Redox Signals Controlled by the Selenoprotein Thioredoxin Reductase 1

**DOI:** 10.3390/cancers14205011

**Published:** 2022-10-13

**Authors:** Chelsey D. Kline, Madeleine Anderson, John W. Bassett, Gail Kent, Rachel Berryman, Matthew Honeggar, Shosuke Ito, Kazumasa Wakamatsu, Arup K. Indra, Philip J. Moos, Sancy A. Leachman, Pamela B. Cassidy

**Affiliations:** 1 Department of Dermatology, Oregon Health & Science University, Portland, OR 97239, USA; 2 Institute for Melanin Chemistry, Fujita Health University, Toyoake 470-1192, Japan; 3 Department of Pharmaceutical Sciences, College of Pharmacy, Oregon State University, Corvallis, OR 97331, USA; 4 Department of Dermatology, Knight Cancer Institute, Oregon Health & Science University, Portland, OR 97239, USA; 5 Department of Pharmacology and Toxicology, University of Utah, Salt Lake City, UT 84112, USA

**Keywords:** thioredoxin reductase 1, MITF, redox signaling, peroxiredoxin, glutathione

## Abstract

**Simple Summary:**

Melanomas and the melanocytes from which they arise are subject to the damaging effects of reactive oxygen species (ROS) from exogenous and endogenous sources. Many attempts have been made to counteract these effects with antioxidant drugs and natural products. Here, we demonstrate that the antioxidant enzyme thioredoxin reductase-1 controls the stability and function of MITF, the master regulator of melanocytes and melanoma. We believe that understanding these phenomena will generate new melanoma treatment and prevention strategies that are far more effective than brute-force approaches that attempt to simply eliminate ROS from vulnerable tissues and tumors.

**Abstract:**

TR1 and other selenoproteins have paradoxical effects in melanocytes and melanomas. Increasing selenoprotein activity with supplemental selenium in a mouse model of UV-induced melanoma prevents oxidative damage to melanocytes and delays melanoma tumor formation. However, TR1 itself is positively associated with progression in human melanomas and facilitates metastasis in melanoma xenografts. Here, we report that melanocytes expressing a microRNA directed against TR1 (TR1^low^) grow more slowly than control cell lines and contain significantly less melanin. This phenotype is associated with lower tyrosinase (TYR) activity and reduced transcription of tyrosinase-like protein-1 (TYRP1). Melanoma cells in which the TR1 gene (*TXNRD1)* was disrupted using Crispr/Cas9 showed more dramatic effects including the complete loss of the melanocyte-specific isoform of MITF; other MITF isoforms were unaffected. We provide evidence that TR1 depletion results in oxidation of MITF itself. This newly discovered mechanism for redox modification of MITF has profound implications for controlling both pigmentation and tumorigenesis in cells of the melanocyte lineage.

## 1. Introduction

Melanoma arises from pigment-producing melanocytes in the skin. The Surveillance, Epidemiology and End Results (SEER) program of the National Cancer Institute estimates that there will be 99,780 new cases of melanoma in the United States and 7650 deaths from the disease in 2022 [1]. New targeted and immune therapies have revolutionized the treatment of metastatic melanoma. These strategies have resulted in an 18% decrease in death rates for patients with Stage IV disease (distant metastases) in the period between 2013 and 2017 [2]. Unfortunately only about 50% of patients respond to treatment with combinations of immune checkpoint inhibitors, which can cost $102,000–$226,000 for a six month course of nivolumab and ipilimumab [3], and are often associated with dangerous side effects [4]. Our strategy for further decreasing the suffering and deaths caused by melanoma is focused on prevention.

In previous studies of selenium as a chemoprevention agent, we showed that exposure to UV radiation suppresses the enzymatic activity of the selenoproteins thioredoxin reductase (TR) and glutathione peroxidases (GPx), and depletes reduced glutathione (GSH) in melanocytes [5,6]. We also found that selenium supplementation, which enhances the catalytic activities of the TRs and GPxs, prevents these phenomena. Our in vivo investigations of selenium included a prevention study using the hepatocyte growth factor (HGF) mouse model of UV-induced melanoma. We treated mice one day before irradiation with a topical source of selenium (selenomethionine (SeMet)) and found that the appearance of tumors was delayed [5]. However, once the tumors formed, the continued application of SeMet caused the melanomas to grow faster. Thus, supplemental selenium decreases the risk for melanoma when provided at the initiation stage of melanomagenesis, but accelerates the growth of early stage tumors. We followed this study with a human tissue microarray analysis that included nevi, primary melanomas, and metastatic melanoma samples [7]. This analysis showed TR1 was elevated in melanomas relative to nevi and that TR1 levels were positively correlated with thickness in primary melanomas. In the same report, we showed that a human melanoma cell line engineered to express a microRNA that targets the TR1 message was reliant on glycolysis for cell survival in vitro and readily formed highly metastatic tumors in a mouse xenograft model. However, when we combined the TR1 knockdown with the pharmacologic blockade of glycolysis, metastases in the mice were eliminated.

The TR1/thioredoxin 1 (TRX1) and GSH -mediated thiol antioxidant systems are widely recognized for their protection of the cell from the deleterious effects of reactive oxygen and nitrogen species (ROS and RNS) and electrophiles [8,9]. The catalytic activities of TR1 and glutathione reductase transfer reducing equivalents from NADPH to their substrates TRX1 and GSH. The TR1/TRX1 system is an essential player in cancer susceptibility. Its roles in redox signaling pathways have recently emerged as critical mechanisms of controlling intracellular signal transduction [10]. TRX1 reduces disulfide bonds in a wide range of proteins including peroxiredoxins, and TRX-interacting protein (TXNIP), as well as apoptosis signaling kinase-1 (ASK-1 or MAP3K5), PTEN, transcription factors, and proteins involved in the regulation of glycolysis, actin cytoskeleton remodeling, protein synthesis, and protein folding [11]. The redox status of critical cysteines in these proteins controls their structure, function, and stability. 

The TR1/TRX1 system is known to influence the ability of transcription factors such as p53 and NF-κB to bind to DNA by suppressing the oxidative modification of cysteine (Cys) thiols in these proteins [12,13,14,15]. However, protein thiol oxidation is often considered a passive process, important only after antioxidant defenses are breached [16]. It is difficult to assign a role in signal transduction to such a phenomenon, given that the reactivity of most protein thiols is relatively low (rate constants in the range of 10^1^–10^2^ M^−1^s^−1^). This problem was resolved by the discovery that the peroxiredoxin (PRX) family of thiol peroxidases plays key roles in the precise spatial and temporal control necessary for redox reactions to participate in signal transduction. The PRXs are signal transducers in “redox relays”, in which redox signals are transferred first from ROS to PRXs, then to protein substrates such as transcription factors [17]. The TR1/TRX1 system [10] catalytically recycles PRXs to their reduced state, which is inactive in redox signaling. Thus, PRXs and the TR1/TRX1 system participate in redox signaling cascades that elicit precisely controlled responses by the cell in a manner analogous to protein kinases [17,18]. Proteins known to be oxidized by PRXs include transcription factors Stat3 [19] and FOXO3 [20] and the melanoma tumor suppressor ASK1 [21]. It is important to note that the amount of TR1 in the cell is reported to be the rate-determining factor for PRX dimer reduction and inactivation of peroxidase activity [10]; this places TR1 at the apex of cellular redox signaling cascades. 

Here, we continue our evaluation of the multifaceted and sometimes paradoxical roles of TR1 with studies in melanocytes. We use the immortalized human melanocyte cell line PIG1 that is engineered to express an anti-TR1 microRNA. Unexpectedly, we found that melanocytes with low TR1 contain significantly less pigment than the control cells. The results of the studies reported here reveal that TR1 plays a crucial regulatory role in melanin production via redox control of MITF, thus providing new insights into the signal transduction pathways regulated by thiol antioxidant systems in cells of the melanocyte lineage.

## 2. Materials and Methods

### 2.1. Cell Culture

PIG1 (ATCC, Cat# CRL-2208, RRID:CVCL S410), an immortalized human melanocyte cell line [22], was engineered with a microRNA targeting *TXNRD1*, the gene that encodes thioredoxin reductase 1 (TR1). We refer to these cells as TR1^low^. We used a synthetic microRNA (miRNA) targeted against the TR1 mRNA sequence (NM_003330) beginning at nucleotide 752 (TGGGACAGAATGATAGAAGCT). The control cells (TR1^ctrl^ cells), were generated with a microRNA directed at a non-eukaryotic gene as previously described [23]. PIG1-derived cells were propagated in medium which consisted of MCDB 153 (Sigma-Aldrich, St. Louis, MO, USA, cat# M7403) supplemented with 4% fetal bovine serum, human transferrin (Roche (MilliporeSigma, Burlington, MA, USA), cat# 10652202001, 1 µg/mL), human insulin (Sigma-Aldrich cat# 91077C, 5 µg/mL), a-tocopherol (Sigma-Aldrich cat# 258024, 1 µg/mL), basic fibroblast growth factor (bFGF, Sigma-Aldrich cat# F0291 0.6 ng/mL), phorbol 12-myristate13-acetate (Sigma-Aldrich cat # P1585, PMA, 8 nM), and bovine pituitary extract (BPE, ThermoFisher, Waltham, MA, USA, cat# 13028014, 14.4 ng/mL) and antibiotics (Thermofisher cat# 15140163 streptomycin/penicillin at 100 µg/mL and 100 U/mL). In some experiments, we removed this medium 24 h after the cells were plated, then cultured the cells for 72 h in what we refer to as minimal medium that contains only the MCDB 153 medium with 4% fetal bovine serum, PMA, and antibiotics. Cells were incubated in a water-jacketed incubator maintained at 37 °C with an atmosphere of 5% CO_2_. 

M14*^TXNRD1+/−^* cells were generated from the parent M14 human melanoma cell line purchased from the National Cancer Institute (NCI, Cat# M14, RRID:CVCL 1395) using Crispr/Cas9 methodology to target exon 9 of *TXNRD1* [24] as described in Supplementary Materials and Methods. 

For details regarding growth curves, immunochemical analysis and Western blot with and without treatment of lysates with NEM, GSH measurements, qPCR analysis, and RNAseq analysis, see Supplementary Materials and Methods. Supplemental Table S1 lists primer sequences.

### 2.2. Immunochemical Analysis and Western Blot

Details in Supplementary Materials and Methods. Antibodies: MITF C5 (Sigma-Millipore, Burlington, MA, USA, Cat# MAB3747, RRID:AB_570596 and Santa Cruz Biotechnology, Dallas, TX, USA, Cat# sc-56725, RRID:AB_784547); MITF (Cell Signaling, Danvers, MA, Cat#97800 RRID:AB_2800289); Thioredoxin 1 TRX1 (Sigma-Aldrich, Cat# HPA047478, RRID:AB_2680063); TR1 (Sigma-Aldrich, Cat# HPA001395, RRID:AB_1858377 and Santa Cruz Biotechnology, Cat# sc-28321, RRID:AB_628405); Prx1 (Thermofisher, Cat# LF-PA0004, RRID:AB_1086614); beta-actin (Cell Signaling, Danvers, MA, USA, Cat# 3700S, RRID:AB_2242334 and Cat# 8457S, RRID:AB_ 10950489); TYRP1 and TYR (Pep1h and Pep7h, gifts from Vincent Hearing, NIH).

### 2.3. 2-D Gel Analysis of Oxidized Protein Complexes

Protocol was adapted from [25]. PIG1 cells were plated, grown to confluence, exposed to 100 μM H_2_O_2_ in PBS for 2 min, and treated with 100 mM NEM for 5 min at room temperature. Cells were scraped from the plate and lysed in buffer containing 40 mM HEPES pH 7.6, 50 mM NaCl, 1 mM EDTA, EDTA-free complete Protease Inhibitor Cocktail tablets (Roche), and 1% peroxide-free Triton X-100. A BCA assay measured protein concentration and 30 µg of protein/well was run under non-reducing conditions on a Bolt 4–12% Bis-Tris gel (Invitrogen NW04120BOX). Protein ladder was added to each well to later visualize in the second dimension, and spaces left between lanes for easier excision. This first electrophoresis was carried out at 60 V to create sharp bands. Upon completion, each lane of interest was excised using a razor blade, ensuring the edges were straight and had tight boundaries. Gel lanes were placed into 5 mL capped tubes with 400 uL 500 mM DTT reducing buffer with protease and phosphatase inhibitors. Tubes were gently rocked at 65 C for 15 min. Gel lanes were rinsed in running buffer and place horizontally on a 2D NuPAGE 4–12% Bis-Tris gel (Invitrogen, NP03266BOX) using the blunt end of a small spatula, ensuring the excised gel fit tightly against the bottom of the well without bubbles. Reducing buffer was layered on top of the slab gel and electrophoresis was carried out at 80 V. The transfer, blocking and staining was carried out under normal conditions. 

### 2.4. Thioredoxin Reductase Activity Assay

The activity of thioredoxin reductases was measured by monitoring a decrease in NADPH as described by Cunniff et al. [10] using selenocystine (Sigma) as a substrate. The assay cannot discriminate between the activity of TR1 and mitochondrial TR3.

### 2.5. Tyrosinase Activity Assay

Tyrosinase activity was measured as described by Lin et al. [26] using L-DOPA as the substrate and measuring the absorbance change at 490 nm. Reaction rates were expressed as absorbance units (AU) per mg of protein. 

### 2.6. Analysis of H_2_O_2_-Induced Protein Oxidation 

PIG1 TR1^ctrl^ and TR1^low^ cells were plated in 6-well plates and treated with 1–100 µM H_2_O_2_ for 1 min. Cells were processed for analysis following a protocol adapted from Li and Kast [27]. See Supplementary Methods for details. 

### 2.7. RNA Extraction and Analysis by qPCR and RNAseq

Total RNA was extracted from cells in culture and purified as described in Supplementary Materials and Methods. Samples were analyzed by qPCR as described using primers shown in Table S1. For RNAseq experiments, RNA was isolated from three different flasks containing one of the two cell lines. DNAse treatment was included in the RNA isolation protocol. Sequencing libraries were prepared using the TruSeq Stranded Protocol with Ribosomal Depletion (Illumina). 

### 2.8. Statistics

Statistical analysis was done on studies that were performed in triplicate or quadruplicate measurements, as noted. Two-tailed Welch-corrected t-tests, and one-way or two-way ANOVA testing, were performed with GraphPad Prism version 7.02. Non-linear curve-fitting was also performed using GraphPad Prism version 7.02. 

## 3. Results

### 3.1. Pigmentation in Melanocytes Is Decreased by TR1 Knockdown

**Figure 1:**
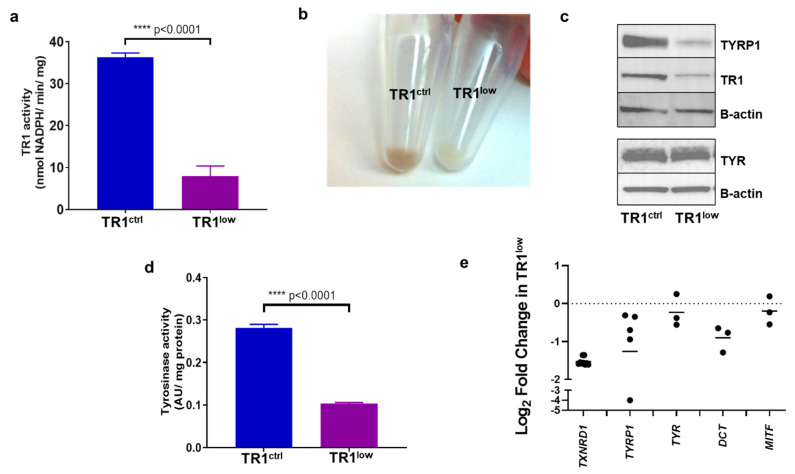
TR1 knockdown melanocytes have decreased pigmentation. (**a**) TR1 activity in TR1^ctrl^ and TR1^low^ cell lines, n = 3. (**b**) Cell pellets from TR1^ctrl^ (left), and TR1^low^ cells (right). (**c**) Western blot analysis of protein expression for TYRP1, TR1, and TYR in TR1^ctrl^ and TR1^low^ cells. (**d**) Tyrosinase activity was measured for the TR1^ctrl^ and TR1^low^ cells, n = 3. (**e**) mRNA analysis by qPCR for TR1^low^ cells relative to TR1^ctrl^ with gene expression normalized to *RPLP0* using the ΔΔC_T_ method. Bars represent the mean log_2_ of the change in gene expression inTR1^low^ cell cells relative to TR1^ctrl^. *p* < 0.001 for *TXNRD1*; *p* = 0.053 for *TYRP1*; *p* = 0.22 for *TYR*; *p* = 0.01 for *DCT*; *p* = 0.45 for *MITF*. All cells evaluated were propagated in complete melanocyte medium then moved to minimal medium for 72 h before analysis (see Section 2). Full Western blot images can be found at Supplementary Materials.

We transformed the immortalized human melanocyte cell line PIG1 [22] with a lentivirus encoding a microRNA that targets the TR1 mRNA, a product of the gene *TXNRD1*. A control PIG1-derived cell line was generated with a lentivirus encoding a microRNA directed at a non-eukaryotic gene [23]. The specific activity of TR1 in the control (PIG1 TR1^ctrl^) cell line was 36.3 nmol NADPH/min/mg protein, significantly higher than the TR1 knockdown cells (PIG1 TR1^low^) cells, which have a specific activity of 7.9 nmol NADPH/min/mg, *p* < 0.0001 (Figure 1a). Residual activity is likely due primarily to the mitochondrial isoform of TR, which is not targeted by the microRNA [7]. The specific activity of TR1 in the PIG1 parent cell line is 26 nmol NADPH/min/mg (Figure 1a). Knocking down TR1 also decreased the growth rate of TR1^low^ cells compared to control cells with doubling times of 2.3 and 1.7 days, respectively (Supplementary Figure S1). Unexpectedly, the cell pellets from TR1^low^ cells were visibly less pigmented than those prepared from TR1^ctrl^ cells (Figure 1b). We assessed the relative abundance of TYR and tyrosinase-related protein 1 (TYRP1) in the two engineered cell lines. In Figure 1c, Western blot analysis shows that TYR protein in TR1^ctrl^ and TR1^low^ cell lines are similar, but TYRP1 is significantly decreased in the TR1^low^ cells. We saw a similar effect when we silenced *TXNRD1* using a mixture of three siRNAs in the M14 melanoma cell line. In these experiments, we found decreased expression of both TYRP1, and TYR (Supplementary Figure S2). We measured tyrosinase activity in cell lysates prepared from the PIG1 cell lines and found that the activity in the TR1^low^ cells was reduced by 64%, *p* < 0.0001 (Figure 1d). 

When we examined the expression of pigmentation genes by qPCR, we found that the transcription of *TYRP1* and *DCT* were consistently reduced in TR1^low^ cells compared to TR1^ctrl^ cells. The transcription of *TYR* and *MITF* trended lower but this was not the case in every comparison that we made (Figure 1e). We also examined the transcription of melanin synthesis genes *TYR, TYRP1, PMEL, MLANA* and *DCT* by RNAseq analysis in M14 human melanoma cells in which we have disrupted *TXNRD1* using Crispr/Cas9 (M14^TXNRD1+/−^ cells, Supplementary Figures S3 and S4). When compared to clones containing intact *TXNRD1*, the M14*^TXNRD1^*^+/−^ cells have dramatically decreased expression of these genes (Supplementary Figure S3).

### 3.2. Alterations in TR1 Activity Affect TYRP1 and MITF Protein Levels as well as Tyrosinase Activity

**Figure 2:**
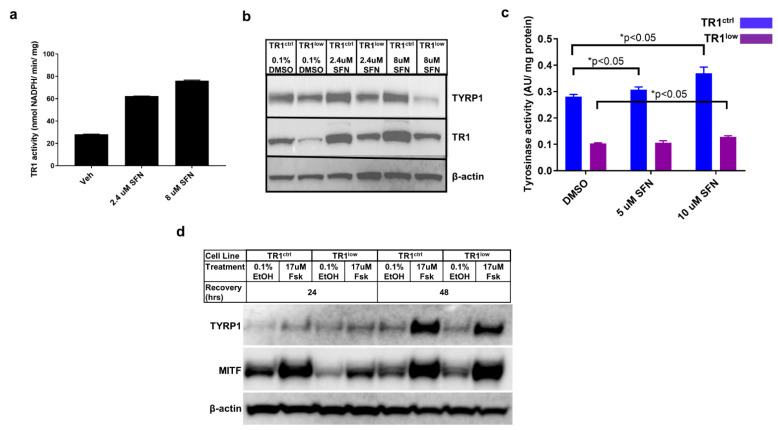
TYRP1 and MITF protein levels, as well as tyrosinase activity are dependent on TR1. (**a**) The small molecule antioxidant sulforaphane (SFN) causes a dose-dependent increase in TR1 activity in PIG1 cells after 24 h treatment. (**b**) Effects of 24 h treatment with SFN on TR1 and TYRP1 protein expression in TR1^ctrl^ and TR1^low^ cells. (**c**) Tyrosinase activity in TR1^ctrl^ (blue) and TR1^low^ (purple) cells after 24 h of treatment with SFN. (**d**) Comparison of the time-dependent effect on protein expression of TYRP1 and MITF after FSK treatment of TR1^ctrl^ and TR1^low^ cells. For each experiment cells were transfered to minimal medium without growth factors (see Section 2) for 72 h before beginning treatments. Full Western blot images can be found at Supplementary Materials.

We have reported that the small molecule antioxidant sulforaphane (SFN) potently induces the expression of TR1 in melanoma cells [7]. We evaluated SFN in our PIG1 parent cells and found that it increased TR1 activity in these cells as well (Figure 2a). In both PIG1 engineered cell lines, treatment with SFN resulted in modest increases in TYRP1 protein (Figure 2b) and TYR activity (Figure 2c). However, TYR activity in the TR1^low^ cells remained substantially lower in all treatment groups. We analyzed the SFN-treated TR1^ctrl^ and TR1^low^ cells for eumelanin (EM) and pheomelanin (PM) content using high-pressure liquid chromatography (HPLC) assay for their degradation products [28,29]. Both EM and PM were significantly decreased in PIG1 TR1^low^ cells compared to PIG1 TR1^ctrl^ cells (Supplementary Figure S5), but SFN had little effect on melanin in either cell line.

We then investigated the effects of TR1 knockdown on the activation of the canonical melanin synthesis pathway. In this and all of the experiments depicted in Figure 1 and Figure 2, melanocyte cell lines were propagated in complete melanocyte medium (see Section 2) then deprived of bovine pituitary extract (BPE) for 48 h before the initiation of the experimental treatments. BPE is a source of α-melanocyte stimulating hormone (α-MSH). The stimulation of pigment synthesis begins by the binding of α-MSH to the melanocortin-1 receptor (MC1R), and activates adenylate cyclase and production of cAMP. cAMP activates protein kinase A (PKA), which in turn phosphorylates the transcription factor cyclic AMP response element binding protein (CREB). CREB binding to the promoter of *MITF* increases production of MITF, the transcription factor and master-regulator of melanin synthesis genes [30]. To generate data shown in Figure 2d, cells deprived of α-MSH were treated with 17 µM forskolin (FSK). FSK is a diterpene natural product used in cell culture and mouse models to stimulate melanin synthesis via increased production of cAMP [31]. At 24 h after treatment, Western blot analysis of protein lysates showed that the amount of TYRP1 was low and approximately equal for all treatments in both cell lines, and expression of MITF was highest in both the vehicle- and FSK-treated TR1^ctrl^ cells. MITF was increased after 48 h of FSK treatment to the same extent in both cell lines, but TYRP1 was still lower in the TR1^low^ cells. Thus, melanocytes deficient in TR1 produce TYRP1 after the stimulation of adenylate cyclase increases MITF, but they respond much more slowly than do the cells with a normal quantity of TR1.

### 3.3. The TR1/TRX1 Network Cooperates with the GSH Network in the Regulation of TYRP1 and MITF

**Figure 3:**
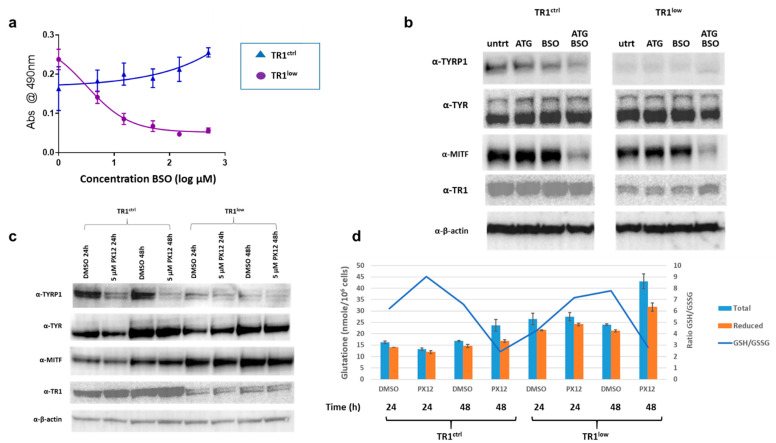
Acute effects of TR1, TRX1 and GSH inhibition on the expression of melanin synthesis proteins. (**a**) Growth of TR1^low^ cells is sensitive to depletion of GSH by 72 h treatment with BSO, while TR1ctrl cells are unaffected (**b**) Western blot analysis comparing expression of TYRP1, TYR, and MITF after 48-h treatment with 10 µM BSO, 60 µM ATG or both BSO and ATG in the TR1^ctrl^ and TR1^low^ cells. (**c**) Western blot analysis comparing protein expression of MITF, TYRP1, and TR1 in TR1^ctrl^ and TR1^low^ cells after 24 and 48 h of 5 µM PX12 treatment. (**d**) GSH was measured in a portion of the cells treated with PX12 in (**c**). The amounts of reduced GSH are increased in PX12-treated TR1^ctrl^ cells and TR1^low^ cells at 48h compared to 24h (*p* < 0.003 for both cell lines). In all treatments GSH in TR1^ctrl^ cells is significantly lower than in TR1^low^ *p* < 0.001. Full Western blot images can be found at Supplementary Materials.

The TR1/TRX1 and GSH thiol antioxidant networks have many overlapping and redundant functions. Knockdown of TR1 activity elicits a compensatory increase in the amount of reduced GSH in many systems [32], despite the tight control of intracellular GSH via competitive inhibition of the rate-limiting enzyme in its biosynthesis, γ-glutamylcysteine synthase (γ-GCS), by GSH itself [33]. Consistent with these precedents, we found increased GSH in PIG1 TR1^low^ relative to TR1^ctrl^ cells [34]. In the TR1^low^ cells, the increase in GSH is likely facilitated by the increase in the expression of genes required for cysteine and cystine transport into the cell (*SLC7A11* and *SLC1A4*) since cysteine is the rate-limiting amino acid required for GSH synthesis (Supplementary Figure S6 and [35]). We examined the acute effects of modulating the TR1 and GSH systems on TYR and TYRP1 expression in PIG1 cells using the small molecule inhibitors aurothioglucose (ATG), a selective inhibitor of TRs [36]), PX12 (a reversible inhibitor of TRX1 [37]), and buthionine sulfoximine (BSO, an inhibitor of γ-GCS). TR1^low^ cells are sensitive to BSO as determined by a tetrazolium dye-based assay (MTS assay, Promega) with an IC_50_ of 3 µM after 72 h of treatment (Figure 3a). In contrast, the TR1^ctrl^ cells were unaffected at concentrations of up to 500 µM. However, 48-h treatment with 10 µM BSO depleted GSH in both cell lines by more than 85%, with no significant cytotoxicity [33]. We used these conditions to assess the effects of GSH depletion on the expression of TYR, TYRP1 and MITF. Western blot analysis showed that in TR1^low^, cells TYRP1 protein was very low under all conditions, but TYR expression was similar to that in the TR1^ctrl^ cells and was unaffected by depletion of GSH (Figure 3b). Treatment with 60 µM of the TR inhibitor ATG (IC_50_ of ~100 µM at 48 h in both cell lines, Supplementary Figure S7a), had no effect in either cell line (Figure 3b). However, treatment with both ATG and BSO decreased TYRP1 in the TR1^ctrl^ cells, and TYR in the TR1^low^ cells. Inhibiting both the TR1 and GSH systems resulted in significant depletion in MITF in both cell lines.

TR1^ctrl^ and TR1^low^ cells have similar sensitivities to the TRX1 inhibitor PX12 with IC_50_ at 48 h of approximately 10 µM (Supplementary Figure S7b). We treated cells with 5 µM PX12, (Figure 3c) and analyzed protein by Western blot. We observed a substantial decrease in TYRP1 in TR1^ctrl^ cells after 24 h with a further decline at 48 h of treatment. TYRP1 was low under all conditions and only modestly decreased by PX12 in the TR1^low^ cells. TYR was unaffected in either cell line at 24 h. TYR and MITF increased in both cell lines in the vehicle (0.1% DMSO) treated cells at 48h relative to the 24 h time point, and this effect was not altered by the drug. This phenomenon is not unexpected since DMSO is known to have numerous effects on both proteins and nucleic acids in cell culture, and has documented antioxidant properties ([38] and references therein). Note that the drugs used in Figure 3b are water soluble therefor the untreated cells are not subject to this vehicle effect. We also tracked the changes in GSH in the PX12-treated cells (Figure 3d). In the TR1^ctrl^ cells, PX12 decreases GSH relative to vehicle control at 24 h, and both cell lines responded to the drug with elevated levels of total and reduced GSH after 48 h treatment. These data are consistent with in vivo and clinical data which shows: (1) PX12 at toxic doses depletes GSH in cells treated in culture [39]; and (2) patients treated with PX12 exhale the malodorous gas 2-butanethiol, which is a product of the reaction of PX12 with reduced thiols including those in GSH and TRX1 [40]. Both GSH and TRX1 are oxidized in the reaction of PX12 and must be recycled by their reductases, and glutathione reductase and TR1, respectively. In our system, GSH rebounds at 48 h after application of PX12 in both cell lines. There is a modest increase in TR1 in the TR1^ctrl^ cells.

The results shown in Figure 3 were replicated for Figure 3b (3 times) and Figure 3c (2 times). However, we did see some differences in the effects of ATG and PX12 from similar experiments performed two years earlier. The most likely source of the variability is the use of different lots of serum. The basal medium contains only 20 nM selenium, and if additional selenium is not supplied by the serum, thioredoxin reductase activity is not maximized [5]. This can make cells more sensitive to ATG and PX12 than they would be under selenium-sufficient conditions. Variability in selenoprotein activity can be avoided by maintaining cells in medium supplemented with sodium selenite at concentrations of up to 500 nM [5,41,42]. Using a different lot of FBS (likely selenium deficient) from that used in the experiments shown in Figure 3, we saw that the TR1^ctrl^ cells produced less TYRP1 after treatment with ATG, but recovered TYRP1 expression after 48 h treatment with PX12 (Supplementary Figure S8). TR1^low^ cells cultured in this same medium were more sensitive to PX12 in that both MITF and TYRP1 proteins were severely depleted after 48 h treatments. Despite these differing results, we confidently conclude that the protein levels of TYRP1 are decreased in both cell lines after genetic or chemical inhibition of the TR1/TRX1 system, and we hypothesize that this effect can be attributed to inhibition of the transfer of reducing equivalents through the TR1/TRX1 system to the molecular complex governing the transcription of TYRP1.

### 3.4. MITF and PRX1 Are Reversibly Modified by H_2_O_2_ and Loss of TR1 in Melanocytes

**Figure 4:**
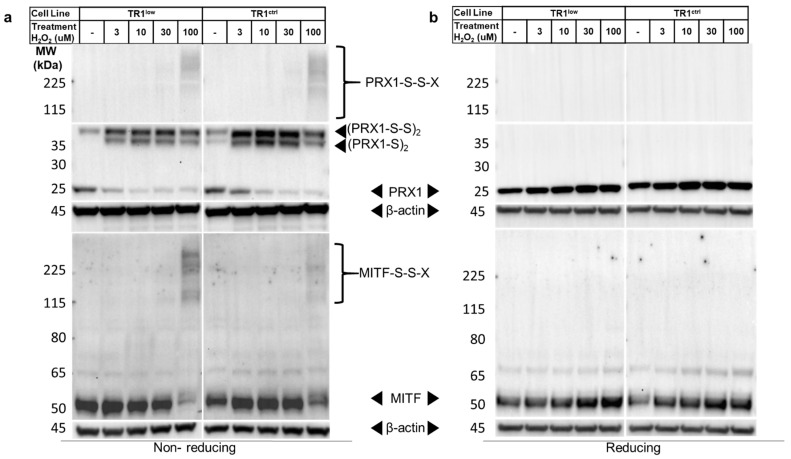
PRX1 and MITF are reversibly modified by oxidation with H_2_O_2_. (**a**) A dose-dependent formation of high-molecular-weight disulfide-mediated complexes that contain PRX1 and MITF (solid arrowheads, (PRX1-S)_2_, (PRX1-S-S)_s_ and MITF-S-S-X) are detected by Western blot in TR1^low^ and TR1^ctrl^ cell lines fractionated under non-reducing conditions after treatment with H_2_O_2_. MITF detected in TR1^low^ cells is increased at baseline relative to TR1^ctrl^ cells. (**b**) Under reducing conditions, both the MITF and PRX1 complexes are transformed into monomers. These results are representative of those from four different experiments. Full Western blot images can be found at Supplementary Materials.

We now turned to the question of the mechanism by which redox signaling mediated by TR1/TRX1 affects MITF and melanin synthesis. To do this we began by assessing the effects of TR1 knockdown on PRX1 oxidation, which is known to affect the redox status of transcription factors and is catalytically recycled by TRX1. PRX1 forms disulfide-mediated homodimers in the presence of both exogenous and endogenous H_2_O_2_ that can then oxidize other proteins in redox signaling cascades. PRX1 dimers are easily detected by Western blot after fractionation of cell lysates by non-reducing SDS-PAGE [43]. We analyzed PRX1 after treating TR1^ctrl^ and TR1^low^ cells with a short (1 min) pulse of 1–100 µM H_2_O_2_. The cells and the lysates were treated with N-ethylmaleimide (NEM) to covalently modify reduced thiols and prevent oxidation artifacts and disulfide shuffling after cell lysis [27]. Interestingly, we found a mixture of PRX1 monomers and dimers in both cell lines at baseline (Figure 4a). Prx1 was detected in the disulfide form ((PRX1-S)_2_ in Figure 4a), or in a slightly less mobile species that is likely a PRX1 dimer with two disulfide bonds ((PRX1-S-S)_2_) [44]. The two-disulfide form of the dimer is the predominant dimeric species in the untreated TR1^low^ cells in the example shown in Figure 4a, but while repeated experiments supported this trend, the difference was not statistically significant. Our analysis of the redox state of bulk TRX1, a major source of reducing equivalents for PRX1, found only a modest increase in oxidized TRX1 in the TR1^low^ cells relative to TR1^ctrl^ (Supplementary Figure S9) [10]. H_2_O_2_ treatment for 1 min increased the ratio of (PRX1-S-S)_2_ and (PRX1-S)_2_ to reduced monomeric PRX1 in both cell lines. Consistent with the redox-mediated nature of these dimers, PRX1 was detected in all treatments at 25 kD only when the lysates were fractionated under reducing conditions (Figure 4b).

When we analyzed the same lysates for MITF, the ratio of the amount of protein detected in the untreated TR1^ctrl^ cells compared to the TR1^low^ cells was 0.51 in the non-reducing gels (*p* = 0.001) and 0.62 in the reducing gels (*p* = 0.01) (Figure 4a,b; box and whisker plots of data from 6 different biological replicates can be found in Supplementary Figure S10). Additionally, in cells treated with 100 µM H_2_O_2_ the MITF band migrating at ~50 kD became very faint on the non-reducing gel, and high molecular weight bands appeared in their place. These blots are very similar to published analyses of other redox-regulated proteins [21,45,46,47] that form disulfide-mediated complexes with other proteins after exposure to ROS. In our work, fractionation of the same lysates under reducing conditions results in the reversion of these high-molecular-weight species to a single band on the Western blot at the predicted molecular weight of MITF (Figure 4b). We hypothesize that these high-molecular-weight bands are disulfide-linked complexes that contain MITF (denoted MITF-S-S-X).

**Figure 5:**
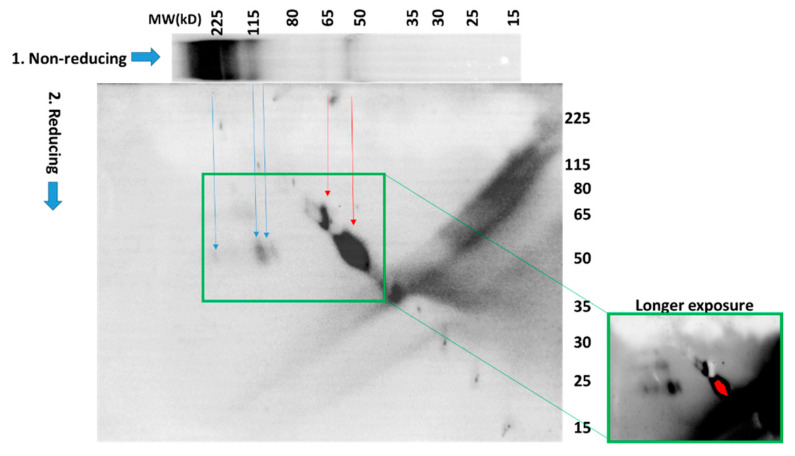
2-D SDS-PAGE analysis under both non-reducing and reducing conditions reveals numerous redox-modified MITF species. PIG1 melanocytes were separated by 2-D electrophoresis as described in the text. Western blot indicated that the high-molecular-weight species are converted to bands that migrate at the apparent molecular weight of MITF after chemical reduction. The majority of the MITF detected migrates at the same or similar molecular weights in both dimensions. Longer exposure (green boxes) shows multiple high-molecular weight MITF-containing species.

**Figure 6:**
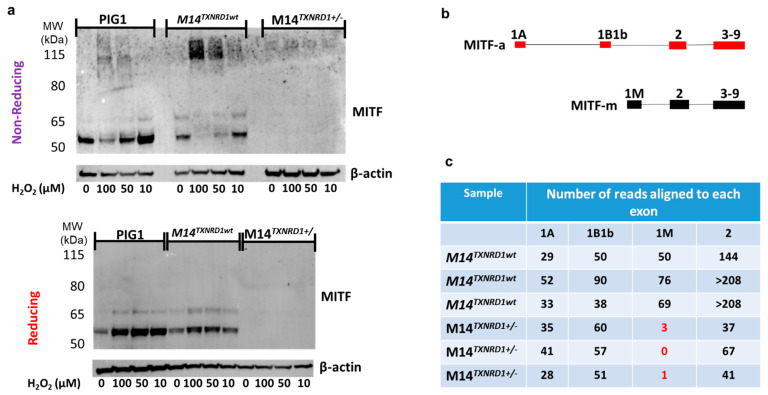
Disruption the gene encoding TR1 (*TXNRD1*) using CRISPR/Cas9 results in loss of expression of the melanocyte-specific isoform of MITF (MITF-M) in melanoma cells. (**a**) Analysis of the PIG1 parent cell line and M14 melanoma cells with wild-type TR1 treated with H_2_O_2_ under reducing and non-reducing conditions gives results similar to those found for the TR1^low^ and TR1^ctrl^ cell lines, but M14^TXNRD1+/−^ cells have no MITF-M (**b**) Map of exons used in MITF-A and the shorter melanocyte specific MITF-m. (**c**) RNAseq analysis shows that M14 cells with wild-type TR1 express exon 1M, while M14^TXNRD1+/−^ cells do not (red text). Both cell lines contain transcripts mapping to exons 1A, 1B1b, and 2. Full Western blot images can be found at Supplementary Materials.

To further investigate the identity of the species labeled MITF-S-S-X, we performed a 2-D gel electrophoresis analysis of proteins in which the separation in the first dimension was performed under non-reducing conditions, and in the second dimension under reducing conditions (Figure 5). Lysates from cells treated with 100 µM H_2_O_2_ were prepared in the presence of NEM to preserve the oxidation state of protein thiols. Proteins were separated in the first dimension under non-reducing conditions by SDS-PAGE. The gel was then cut into single-lane strips, with one used for immediate Western blot visualization of the 1st-dimension fractionation (Figure 5, 1.-Non-reducing). Control cells not treated with H_2_O_2_ were also included in this 1-D Western blot, and as expected, did not contain high-molecular weight MITF-S-S-X species (Supplementary Figure S11). In the 2-D analysis, proteins were reduced by treatment with 500 mM dithiothreitol (DTT) at 95 ⁰C. The gel strip was then loaded into the well of a second gel and proteins migrating out of the first gel under reducing conditions were separated by electrophoresis. After transfer of proteins to a polyvinylidene difluoride (PVDF) membrane the blot was probed for MITF (Figure 5). As we expected, the results confirmed the presence of MITF in higher-molecular-weight species (blue arrows, ~225kD and at least 2 at ~115kD) from which a protein that migrates after chemical reduction in the second dimension at a molecular weight consistent with MITF. However, we did not anticipate that the majority of the protein detected (red arrows) migrated in *both dimensions* at or near the expected molecular weight(s) of MITF (both MITF-M, the melanocyte-specific form, and a longer form MITF-A, expressed in melanocytes and other cell types (see Figure 6b)). This could result from the reversible modification of MITF with a low-molecular-weight species after treatment with H_2_O_2_. However, this explanation also requires that the modification masks the epitope recognized by the antibody, rendering the protein undetectable by immunochemical means.

A second lane from the 1-D gel was analyzed in the same manner and the membrane was probed for PRX1. Results from these analyses (Supplementary Figure S12) confirmed that the higher-molecular-weight species labeled (PRX1-S)_2_ and (PRX1-S-S)_2_ in Figure 4 migrate at the molecular weight of monomeric PRX1 in the second (reducing) dimension.

### 3.5. Disruption of TXNRD1 in Melanoma Cells Using CRISPR/Cas9 Results in Loss of Expression of the Melanocyte-Specific Isoform of MITF (MITF-M)

In order to make certain that the redox-mediated phenomena we observed in Figure 4 were not restricted to the engineered PIG1 cells, we performed the same analysis in the parent cell line, in M14 melanoma cells, in M14 melanoma cells transfected with HA-FLAG-tagged MITF, and in M14 cells in which one allele of the gene encoding TR1 (*TXNRD1)* was disrupted using CRISPR/Cas9. Most of our results supported those shown in Figure 4 (Figure 6a and Supplementary Figures S13 and S14). However, in the M14^TXNRD1+/−^ cell line in which the gene encoding TR1 was disrupted using CRISPR/Cas9, no MITF bands were detected (Figure 6a). RNAseq analysis of M14^TXNRD1+/−^ and M14^TXNRD1wt^ cell lines provides critical information that allows us to interpret this result. MITF-M is encoded by an mRNA that includes exon 1M; this mRNA is produced only in cells of the melanocyte lineage [47]. M14^TXNRD1+/−^ cells do not produce RNAs that map to exon 1M (Figure 6c and Supplementary Figure S15). mRNA from M14 cells with only wild-type TR1 yields reads that align to exons 1A, 1B1b, and 1M in MITF. Exons 1A and 1B1b are included in the mRNAs that encode MITF isoforms expressed in a variety of cell types, including melanocytes. Proteins translated from these and the melanocyte-specific mRNAs are visible at ~65 and 52 kD, respectively, in the PIG1 and M14 cells. The MITF C5 antibody used in these experiments was raised to a peptide in the amino terminus of MITF common to all human MITF isoforms [48,49]. The M14^TXNRD1+/−^ cells produce RNAs that map to exons 1A and 1B1b, but in amounts estimated to be only about 35% of those in the wild-type cells based on relative numbers of reads mapping to exon 2. Together, the data in Figure 4, Figure 5 and Figure 6 lead us to conclude that: (1) redox modifications of cysteine thiols in the MITF are caused by H_2_O_2_ and that these modifications may be reversed or prevented by TR1; and (2) production of mRNA that includes the melanocyte-specific exon 1M requires TR1 in M14 melanoma cells.

## 4. Discussion

The major finding of our work is that TR1 is involved in the redox control of the activity and stability of the transcription factor MITF. TR1 transfers reducing equivalents from NADPH to TRX1, which in turn reduces disulfide bonds in a wide range of proteins, including transcription factors [11]. Transcription factors are reduced either directly by TRX1 or via the provision of reducing equivalents to other reductases including the peroxiredoxins (PRXs). The redox status of critical cysteines in transcription factors controls the structure and function of these proteins, including interactions with other proteins and DNA. For example, in the p50 subunit of the transcription factor NF-κB, oxidation of Cys^62^, which can be reversed by TRX1, prevents binding of the protein to DNA [50]. In our work reported here, we provide evidence that the transcriptional activity of MITF is also affected by the TR1/TRX1 system. The promoters of *TYR* and *TYRP1* contain a binding motif recognized by MITF-M, the M-box [51]; however, they differ in their 5′-enhancer sequences. We observed that the amount of TYRP1 protein, as well as TYR activity, were much lower in the lysates of TR1^low^ melanocytes. The decrease in TYR activity is likely due to the decline in TYRP1 protein production, because the amount of TYR protein that we detect in both cell lines is not significantly affected TR1 knockdown, or by any of the conditions we examined except use of DMSO as a vehicle (Figure 3). Our observations are consistent with the findings of a several laboratories who showed that TYRP1 stabilizes TYR, and TYRP1 enhances its dopa oxidase activity of TYR when the two enzymes are incubated together in vitro using the same assay that we performed in Figure 1d ([52] and references cited therein). We conclude that decreased TYR activity in lysates of TR1^low^ melanocytes is a consequence of low TYRP1 levels, and that TYRP1 and TYR are differentially regulated at the level of transcription as a consequence of TR1 knock down. We believe that our data in Figure 4 are consistent with involvement of redox modification of MITF in the difference between transcription of *TYRP1* and other MITF-regulated genes. However, we cannot rule out an indirect effect of an unknown mediator on non-redox alterations such as phosphorylation, to MITF or other cofactors that regulate *TYRP1* transcription.

We see a more dramatic effect on the expression of melanin synthesis enzymes in M14 melanoma cells in which we have disrupted the gene encoding *TXNRD1* using Crispr/Cas9 (Figure S3 and Figure 6c). The cell line used for RNAseq experiments was one of 3 clones containing heterozygous deletions in exon 9 of *TXNRD1* in which we detected no MITF by Western blot. RNAseq analysis revealed that the *TXNRD1*^+/−^ cells lost expression of the melanocyte-specific exon 1M of *MITF* as well as additional MITF target genes related to the synthesis of melanin (*TYR*, *TYRP1*, *PMEL*, *MLANA,* and *DCT)*. A recent report demonstrated that MITF regulates the expression of *SOX10* [53], and our RNAseq data shows that *SOX10* is also significantly depleted in the *TXNRD1*^+/−^ melanoma cells (less than 1% that of the *TXNRD1^wt^* cells, p_adj_ = 1.2 × 10^−10^). SOX10 and PAX3 are also known to regulate expression of the melanocyte-specific isoform of *MITF* [54], so the loss of MITF-M could be related to loss of expression of *SOX10* (*PAX3* expression is unaffected in M14 *TXNRD1*^+/−^ cells). Schlierf et al., [55] showed in 2002 that SOX10 is redox sensitive in a study that found that a conserved Cys (Cys 71) regulates the dimerization of SOX10 as well as its transactivation of target genes. PAX3 has no reported sensitivity to changes in redox conditions; however, a closely-related family member, PAX8, contains two highly conserved cysteines, Cys 45 and Cys 57, [56] that must be reduced for the protein to bind to DNA [57]. Therefore, the loss of MITF-M in M14 *TXNRD1*^+/−^ cells might also be due to redox-mediated dysfunction of PAX3. MITF-M, in cooperation with Lef1, also regulates its own gene transcription in M14 cells [58,59], and this, in turn, could result in differential regulation of gene expression downstream of MITF-M that we observe in the *TXNRD1*^+/−^ melanoma cells. These possibilities are now under investigation in our labs.

Inhibiting thioredoxin reductases (TRs) in vivo can have results similar to those that we observed in cell culture. Gao et al. treated zebrafish embryos auranofin (AF), a thiogold compound similar to ATG that is also an inhibitor of TRs. They found that the treatment of embryos with 10 µM AF at 72 h post-fertilization caused severe hypopigmentation in addition to developmental defects [60]. They also report that AF treatment reduced *mitfb* and *trp1a* mRNA in a dose-dependent manner. These results align with ours and suggest that TR1 inhibition disrupts melanogenesis by regulation of MITF and the expression of *TYRP1*.

We examined the effects of pharmacologically increasing TR1 protein and enzymatic activity by treating melanocytes with SFN (Figure 3a–c). SFN treatment resulted in a 2-3-fold increase in TR1 protein levels and a 24–31% increase in tyrosinase activity in both the TR1^ctrl^ and TR1^low^ cells. The induction of *TXNRD1* by SFN is mediated by the transcription factor Nuclear Factor Erythroid 2-Related Factor 2 (NFE2L2 or NRF2). NRF2 increases expression of numerous other genes including those encoding the antioxidant enzymes heme oxygenase 1 (HO-1), catalytic and modifier subunits of γ-glutamylcysteine lygase (GCLC and GCLM), thioredoxin, and NAD(P)H quinone dehydrogenase 1 (NQO1) [61,62]. Over expression of NQO1 in acral melanomas and human melanocytes is reported to increase tyrosinase activity [63], but the extent to which this and other antioxidant enzymes contribute to the effects of SFN on pigment cells has not been determined.

We performed protein analyses designed to detect the effects of TR1 loss on redox modifications to the MITF protein itself, and found quite unexpectedly that higher amounts of MITF were detected in untreated TR1^low^ cells, compared to untreated TR1^ctrl^ cells after alkylation of reduced protein thiols with NEM and analysis by Western blot (Figure 4a, no H_2_O_2_ treatment)_._ We propose that this is reasonable if we consider that oxidation of protein thiols in the TR1^low^ cells will prevent reaction with NEM [27]. The reduced thiols in TR1^ctrl^ cells will be modified by NEM, and this could decrease the antibody’s affinity for MITF in the TR1^ctrl^ cells. Glineur et al. reported a similar disruption of protein–protein interactions [64] in a study where they found that modification of a cysteine residue in the c-Rel subunit of NF-κB by NEM prevented phosphorylation of the protein by kinases.

The 2-D analysis of H_2_O_2_-treated melanocytes confirmed that the high-molecular-weight species identified as MITF-S-S-X in the non-reducing Western blot shown in Figure 4a, contains bands that migrate at the molecular weight of monomeric MITF in the second (reducing) dimension. However, we were very surprised to see that most of the MITF detected in the 2-D gel was on the diagonal; it migrated at approximately the same molecular weight in both dimensions. This is in contrast with the results of the 1-D non-reducing gel in which very little MITF is detected at the predicted molecular weight of MITF (~50kD). A plausible explanation for this observation is the rapid and reversible formation of disulfide adducts with small-molecular-weight thiols such as GSH or hydrogen sulfide [65]. The persulfide adducts of cysteine and hydrogen sulfide can undergo further oxidation to negatively-charged perthio-sulfenic, -sulfinic and -sulfonic acids. All such MITF mixed disulfides, which can be disrupted under reducing conditions, should have a molecular weight very close to that of unmodified MITF, and could also interfere with the detection of MITF by antibodies due to the negative charge they introduce to the protein. We immunoprecipitated GSH-modified proteins from both the control and H_2_O_2_-treated PIG1 melanocytes. The glutathionylated proteins were analyzed by Western blot under reducing conditions (which removed the GSH, see Supplementary Figure S16). This study indicated that glutathionylated MITF is formed in both control and oxidized melanocytes at modest (~6% of total MITF) but identical amounts in both control and oxidized melanocytes. Since glutathionylated MITF levels are not affected by H_2_O_2_-treatment, its detection in melanocytes cannot explain the apparent disappearance of MITF from the oxidized protein lysates under non-reducing conditions and its re-appearance on the diagonal in the 2-D analysis after treatment with DTT. Another possible modification of cysteines in MITF is the recently described mono-ADP ribosylation (MARylation) [66]. MARylated cysteine is much more stable chemically than other MARylated amino acid side chains such as glutamate and aspartate. This is consistent with our observation that extremely high concentrations of DTT, (250–500 mM) are necessary to make modified MITF detectable by its antibody. Identifying the modification that underlies this phenomenon will require mass spectrometric analysis.

ROS- and cysteine-thiol-mediated redox signaling regulate the functions of many transcription factors including Egr-1, NF-κB, HIF1-α, CREB, AP-1, FOXO4, Stat 3 and members of the PAX family. Transcription factors are maintained in their reduced states in the nucleus by the dual function DNA-repair enzyme/reductase APE-1/Ref-1 ([67] and reviewed by Marinho et al. [18]). Oxidized APE-1/Ref-1 is dependent on reduction by TRX1 for its catalytic activity [68]. There are 10 cysteine residues in MITF-M (or 9 depending on the splicing of 18 base pairs in exon 6 [69,70]). Melanocytes in the skin are subject to periodic oxidative stress from UV radiation and are exposed to ROS produced during melanin synthesis [71]. Therefore, it seems logical that MITF-M, which resides primarily in the nucleus, might be subject to redox regulation by ROS as well as the TR1/TRX1 system. However, oxidation of proteins rarely occurs via direct reaction of cysteine residues with ROS, but rather by transferring oxidizing equivalents from cellular peroxidases such as PRX1 [18] to target proteins. Oxidized PRX1 dimers such as those we observed (Figure 4) are known to transmit redox signals to other proteins, including transcription factors [21,45]. Unfortunately, the available crystal structures of MITF [72,73] do not allow us to investigate the accessibility of cysteines in MITF or their potential roles in structurally important intramolecular disulfide bonds. These analyses were performed using a polypeptide corresponding to residues 180–296 that includes only the basic, helix-loop-helix and leucine zipper (bHLHZip) domains common to all MITF isoforms. The amino termini of the full-length transcription factors are not included. Furthermore, while some of the peptides co-crystallized with oligonucleotides containing MITF-M binding sites (E-box, M-box, and CLEAR-box) do have the one cysteine residue that is encoded by the long-splice form of exon 6, the region containing that cysteine (Cys188) is not visible in the solution to the crystal structure [72]. Describing the details of how the protein structure of MITF dictates its redox sensitivity will require mass spectrometry-based disulfide mapping of MITF isoforms [74] as well as analyses of oxidized MITF-M and its redox binding partners (the “X” in MITF-S-S-X, Figure 4a).

## 5. Conclusions

In this study, we identify a previously unknown redox regulatory mechanism that controls the function of MITF, a transcription factor that plays a critical role in both pigmentation and melanoma [75,76,77]. Our data reveal that the TR1/TRX pathway regulates MITF and melanin synthesis through a thiol redox network that, when deleted or inhibited, causes changes in the abundance, function, and redox sensitivity of the melanocyte-specific isoform of MITF. In earlier studies, we showed that increasing the activities of thioredoxin reductase and other selenoproteins protected melanocytes from UV-induced oxidative stress, but also increased growth of tumors in a mouse model of UV-induced melanoma [5]. We then showed that TR1 is positively associated with progression in human melanomas, and dramatically increases metastasis of melanoma xenografts [7]. Others have since demonstrated that treatment with antioxidants (N-acetylcysteine or vitamin E), or elevated levels of the antioxidant selenoprotein GPX4 can exacerbate tumorigenesis and facilitate metastasis in mouse models [78,79]. Understanding the relationship between normal redox regulatory mechanisms in the melanocyte and the “double-edged sword” of oxidative stress in UV-irradiated skin and melanoma [80], will expedite the identification of new therapeutic targets for the prevention and treatment of melanoma.

## Data Availability

RNA-Seq data were deposited in the Sequence Read Archive (SRA) under BioProject PRJNA798713. Any additional data is available from the corresponding author upon reasonable request.

## Supplementary Materials

The following supporting information can be downloaded at: https://www.mdpi.com/article/10.3390/cancers14205011/s1, Supplementary Methods. Deletion of TXNRD1 using Crispr/Cas9. Protein analysis and Western blot without N-ethylmaleimide (NEM) treatment. Analysis of H_2_O_2_-induced protein oxidation. RNA Isolation for cDNA synthesis, and Quantitative PCR. Supplementary Table S1. Primers for qPCR. RNA Sequencing. Measurement of Glutathione. Knockdown of TR1 using transient transfections with siRNAs directed against TR1. Immunoprecipitation of glutathionylated proteins and detection of MITF. Supplementary Figures: Supplementary Figure S1. Growth curves and doubling times for TR1ctrl and TR1low cells. Supplementary Figure S2. Knockdown of TR1 using siRNAs directed against TR1. Supplementary Figure S3. RNAseq, Western blot, and TR1 activity in M14 cells wherein TXNRD1 is disrupted using Crispr/Cas9. Supplementary Figure S4. Sequence of M14TXNRD1+/− cells. Supplementary Figure S5. Melanin quantification. Supplementary Figure S6. qPCR analysis of genes related to GSH synthesis in PIG1 TR1low and TR1ctrl cells. Supplementary Figure S7. Cell viability determined by MTS assay after chemical inhibition of GSH, TR1, and TRX. Supplementary Figure S8. Effects of different serum lots on TYR and TYRP1 protein in cells treated with ATG and PX-12. Supplementary Figure S9. PIG1 TR1^ctrl^ and TR1^low^ cell lines have similar TRX1 oxidation states as measured by protein electrophoretic mobility shift assay (PEMSA). Supplementary Figure S10. Box and whisker plot of the ratio of MITF detected in TR1ctrl and TR1low cell lysates treated with NEM. Supplementary Figure S11. 1D immunoblots of MITF and PRX1 from the lysates and gel used to produce the 2D blot shown in Figure 5. Supplementary Figure S12. 2D analysis of PRX1 in PIG1 cells treated with H_2_O_2_ after separation under non-reducing SDS-PAGE in the 1st dimension and reducing conditions in the second dimension. Supplementary Figure S13. Transfection of M14 melanoma cells with a plasmid encoding HA-FLAG-tagged MITF-M and treatment with H_2_O_2_; Western blot under non-reducing conditions. Supplementary Figure S14. Transfection of M14 melanoma cells with a plasmid encoding HA-FLAG-tagged MITF-m and treatment with H_2_O_2_; Western blot under reducing conditions. Supplementary Figure S15. Alignment of RNAseq reads from M14 engineered cells to the genomic sequence of MITF. Supplementary Figure S16. Immunoprecipitated gluthathionylated MITF from PIG1 melanocytes. References [27,81] are cited in the supplementary materials.
