# Supplementary material for: MITF Is Regulated by Redox Signals Controlled by the Selenoprotein Thioredoxin Reductase 1
Source: Cancers (Basel). 2022 Oct 13;14(20):5011. doi: 10.3390/cancers14205011 (PMC9600194; doi:10.3390/cancers14205011)

Figure 1c. Western blot of TR1^{high} and TR1^{lo} cells treated as shown with sulforaphane (SFN) or vehicle (DMSO).
Red box indicates the lanes that were included in Figure 1c.

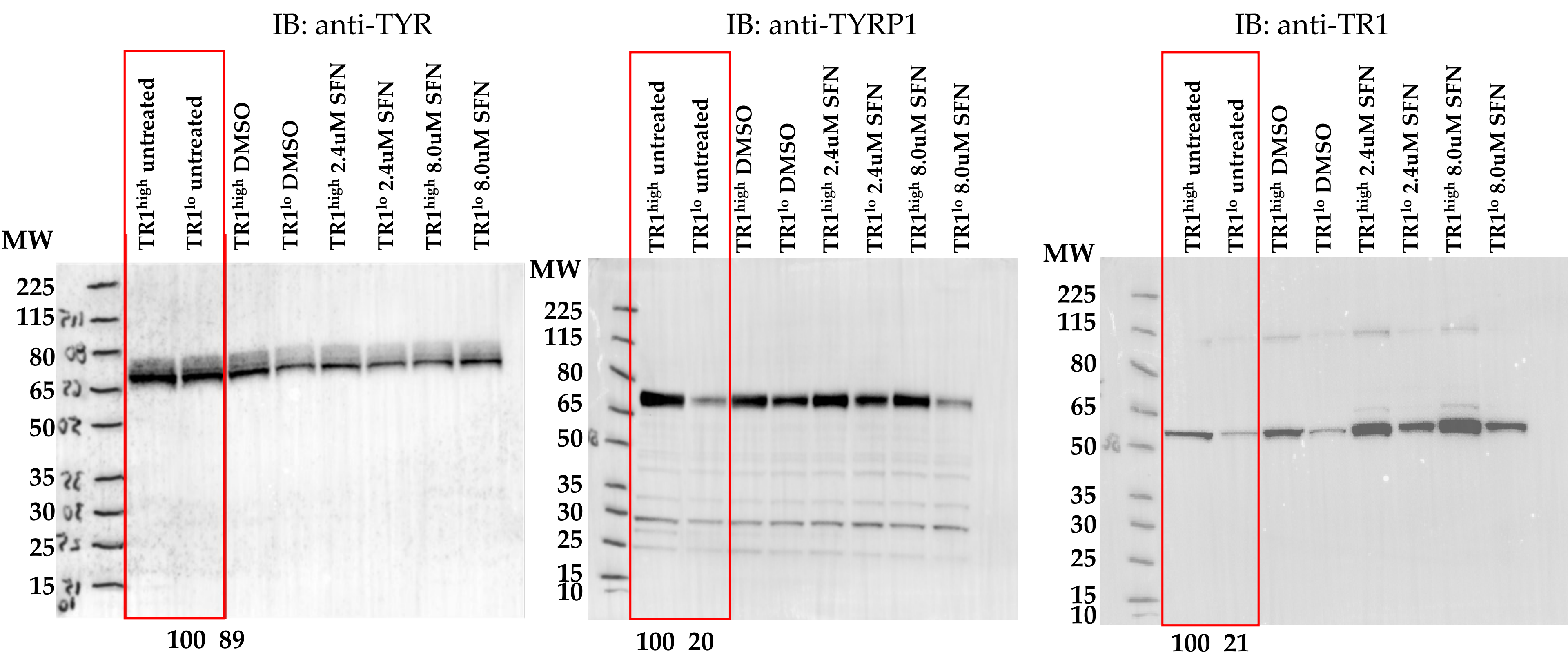

Figure 1c. (continued). Western blot of TR1^{high} and TR1^{lo} cells treated as shown with sulforaphane (SFN) or vehicle (DMSO). Red box indicates the lanes that were included in Figure 1c.

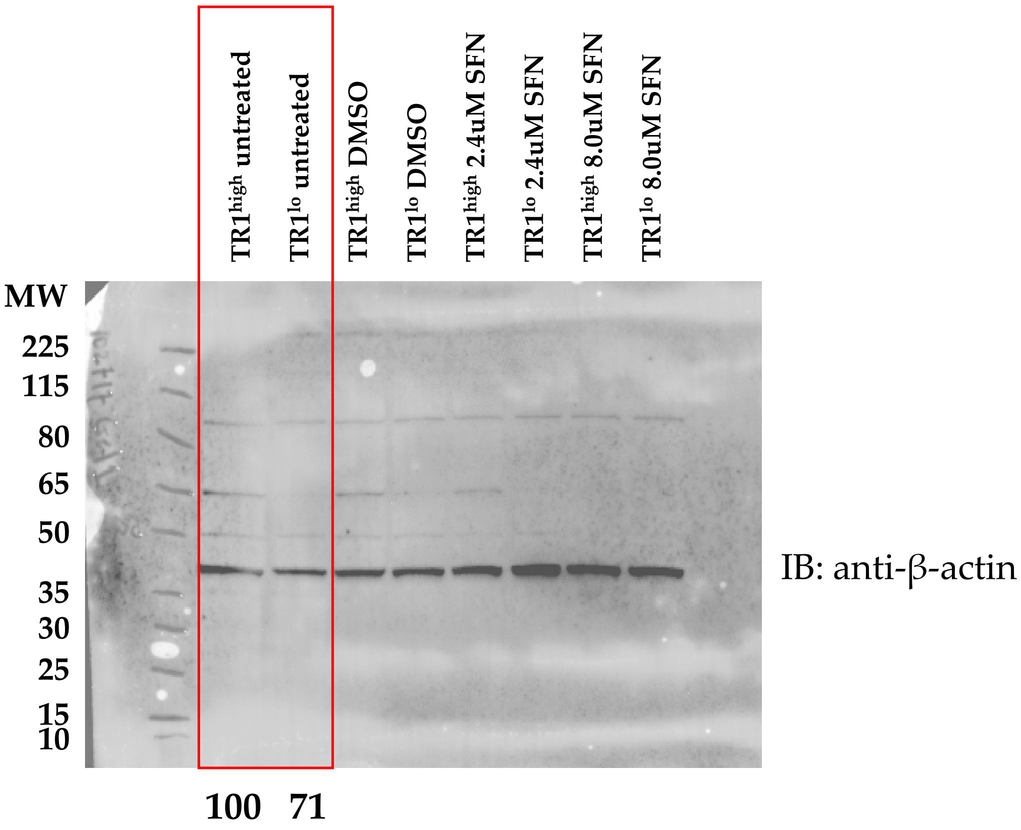

Figure 2b. Effects of 24 hour treatment with SFN on TR1 and TYRP1 protein expression in TR1[high] and TR1[lo] cells. Note the first two lanes (untreated) were used in Figure 1c.

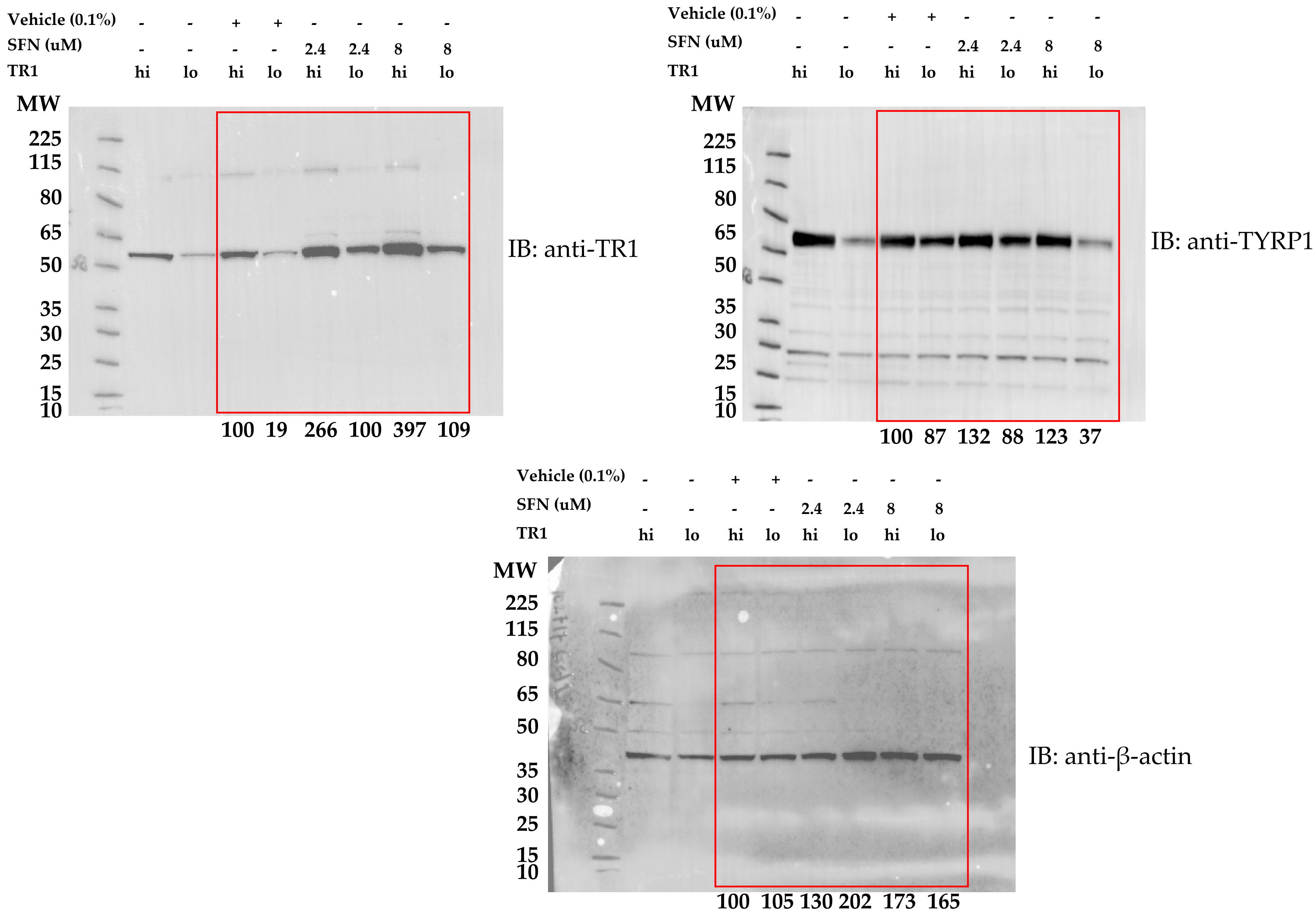

Figure 2d. Comparison of the time-dependent effect on protein expression of MITF after FSK treatment of TR1^{high} and TR1^{lo} cells.

Cell Line	TR1^{high}		TR1^{lo}		TR1^{high}		TR1^{lo}	
Treatment	0.1% EtOH	17uM Fsk	0.1% EtOH	17uM Fsk	0.1% EtOH	17uM Fsk	0.1% EtOH	17uM Fsk
Recovery (hrs)	24				48			

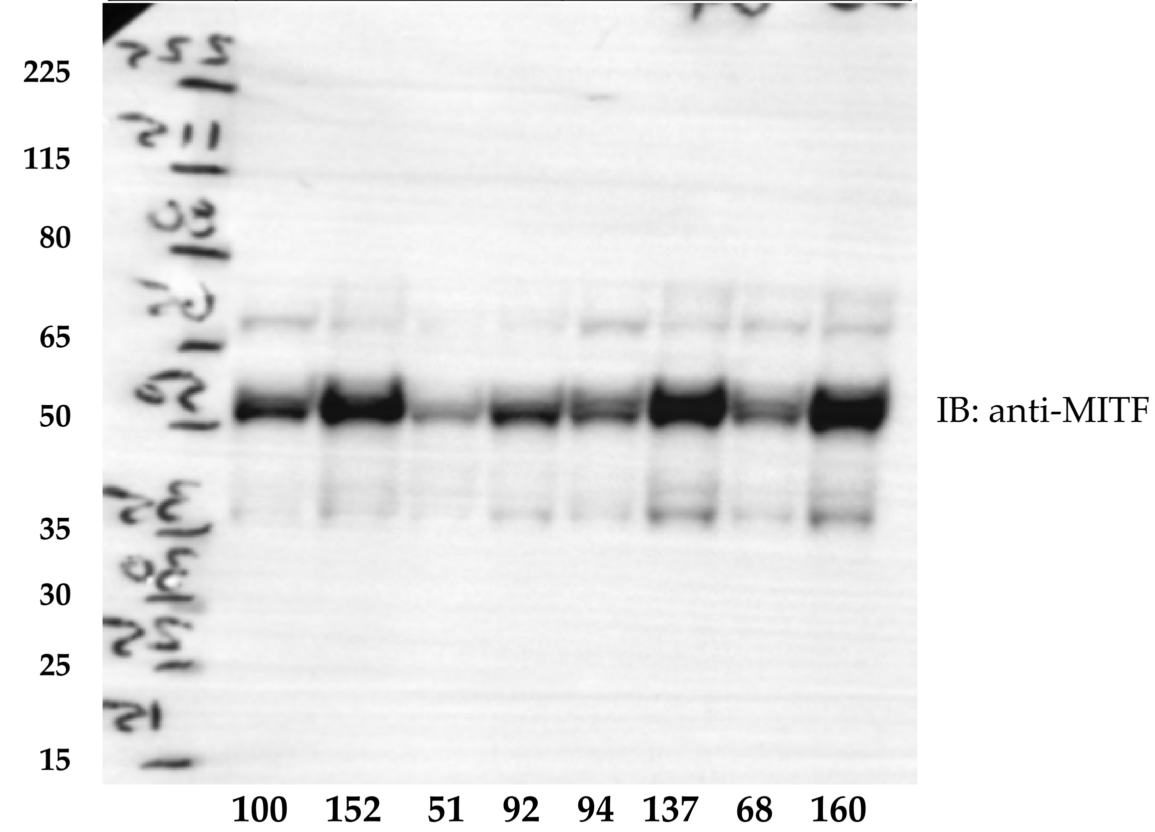

Figure 2d. Comparison of the time-dependent effect on protein expression of TYRP1 after FSK treatment of $TR1^{high}$ and $TR1^{lo}$ cells.

Cell Line	$TR1^{high}$		$TR1^{lo}$		$TR1^{high}$		$TR1^{lo}$	
Treatment	0.1% EtOH	17uM Fsk	0.1% EtOH	17uM Fsk	0.1% EtOH	17uM Fsk	0.1% EtOH	17uM Fsk
Recovery (hrs)	24				48			

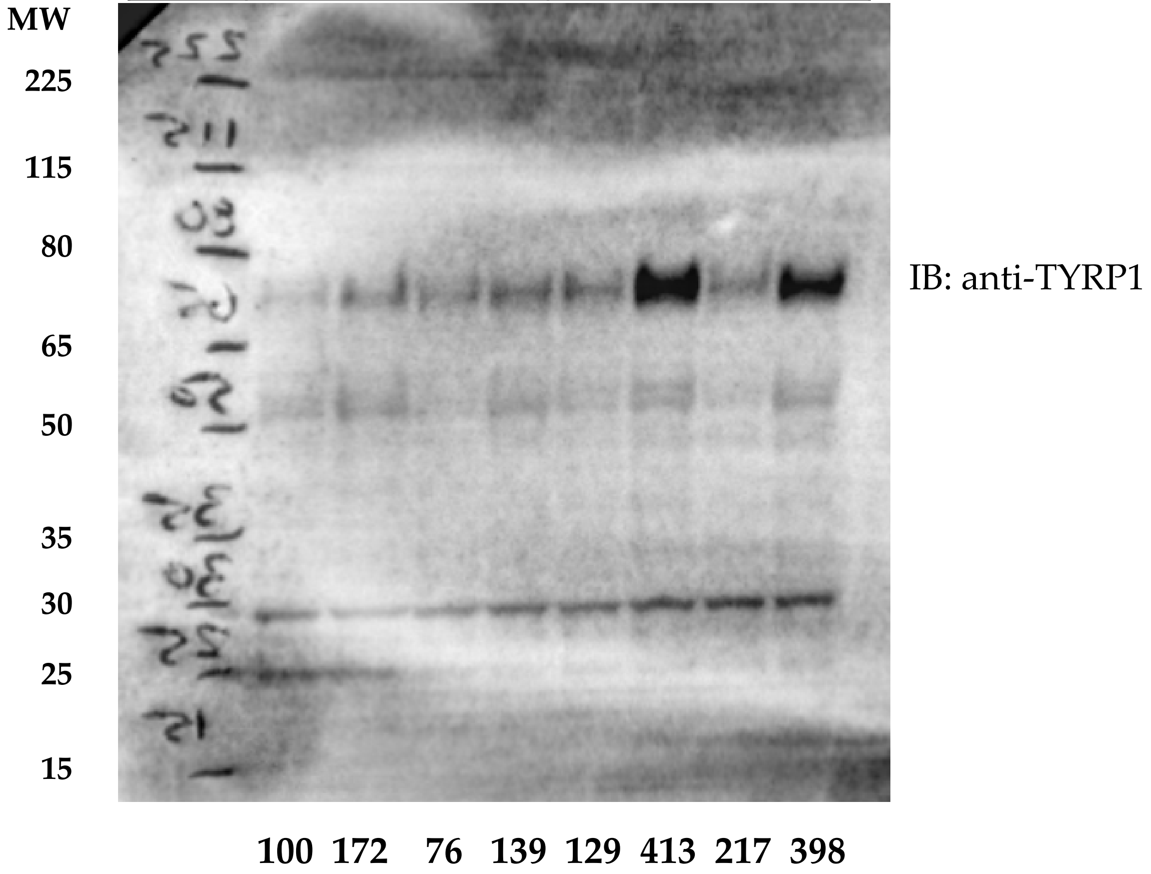

Figure 2d. Comparison of the time-dependent effect on protein expression after FSK treatment of TR1^{high} and TR1^{lo} cells. β-actin probed after TYRP1 with no stripping.

Cell Line	TR1^{high}		TR1^{lo}		TR1^{high}		TR1^{lo}	
Treatment	0.1% EtOH	17uM Fsk	0.1% EtOH	17uM Fsk	0.1% EtOH	17uM Fsk	0.1% EtOH	17uM Fsk
Recovery (hrs)	24				48			

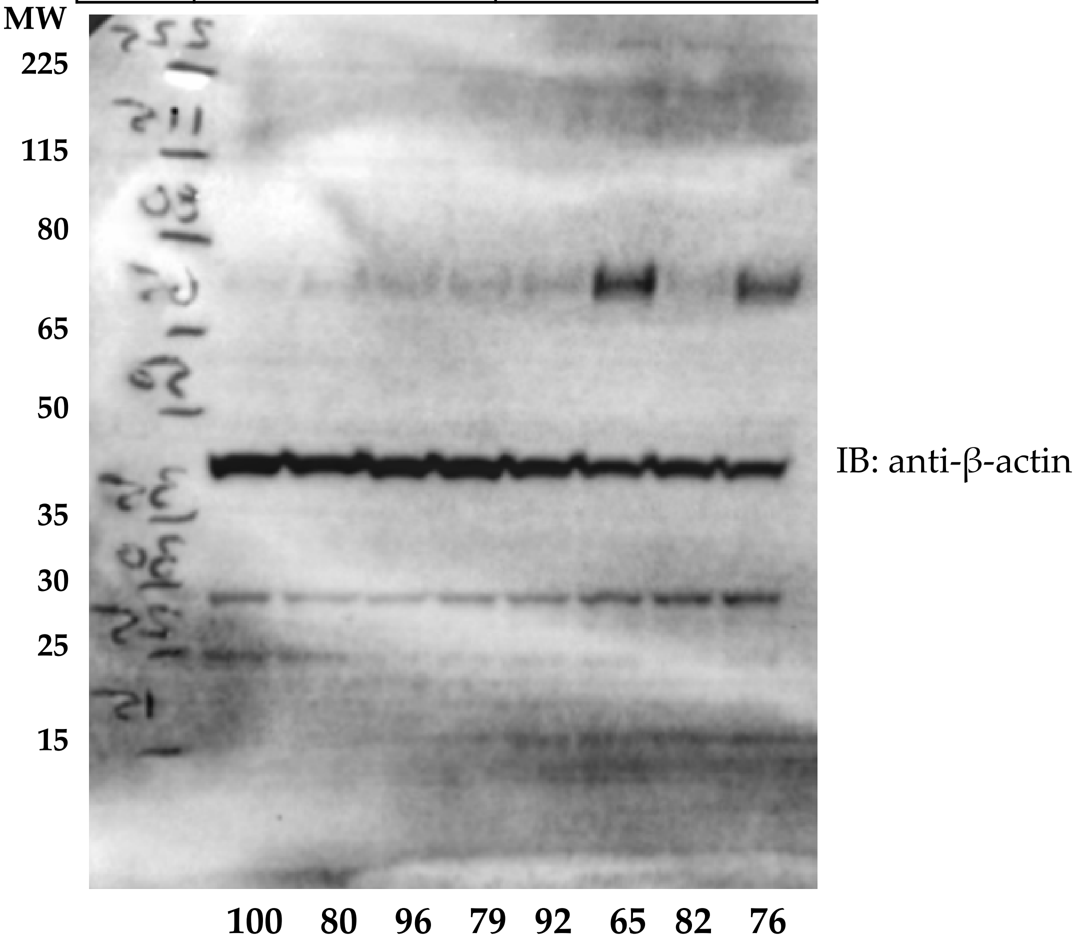

Figure 3b Western blot analysis comparing expression of TYRP1, TYR, and TR1 after 48-hour treatment with 10 μM BSO or 60 μM ATG or both in the TR1^{high} and TR1^{lo} cells. TYRP1 shown here. Note that positive control lanes (*) are from an unrelated experiment and do not appear in the figure.

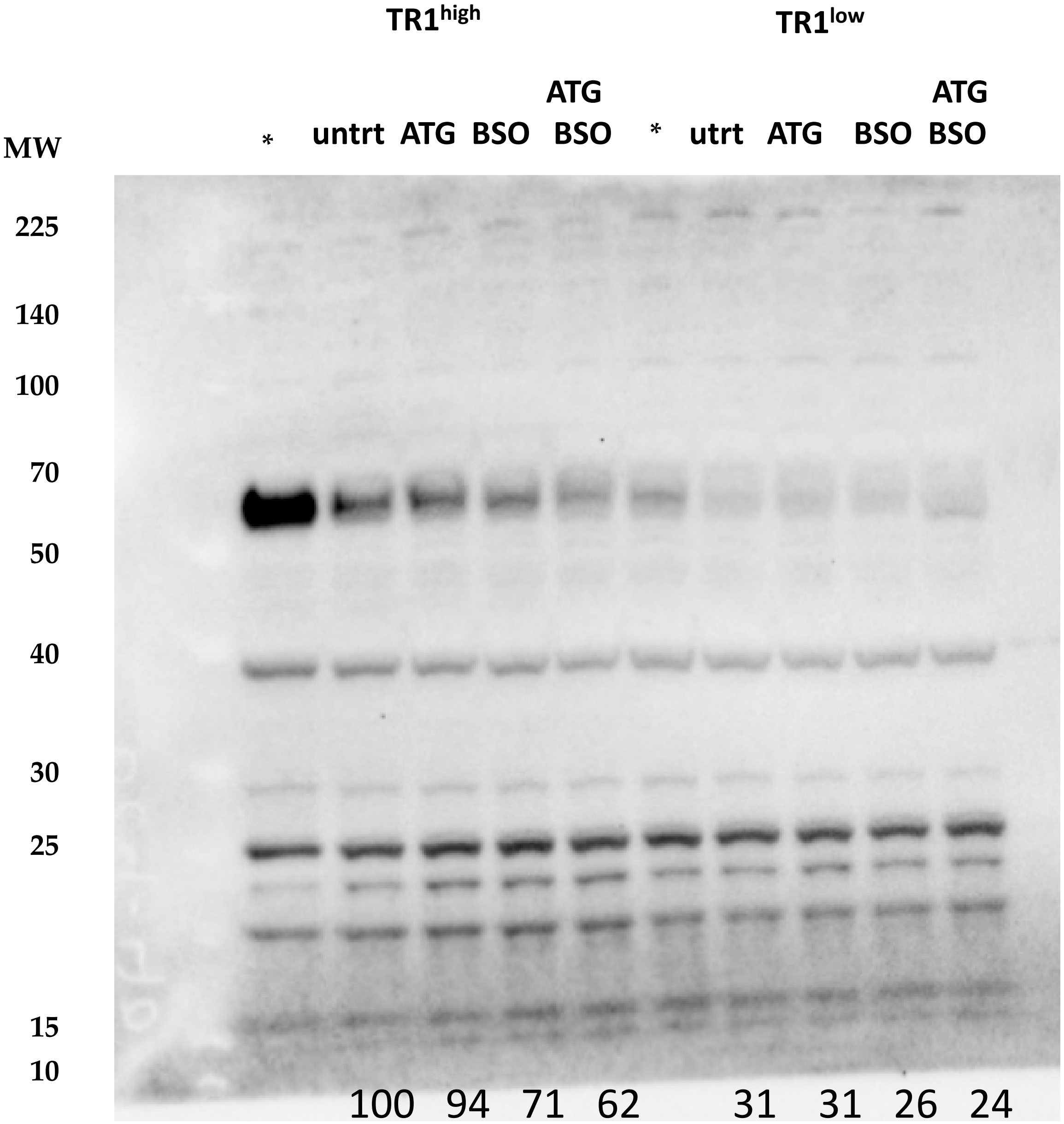

Figure 3b Western blot analysis comparing expression of TYRP1, TYR, and TR1 after 48-hour treatment with 10 µM BSO or 60 µM ATG or both in the TR1^high and TR1^lo cells. TYR shown here. Note that positive control lanes (*) are from an unrelated experiment and do not appear in the figure.

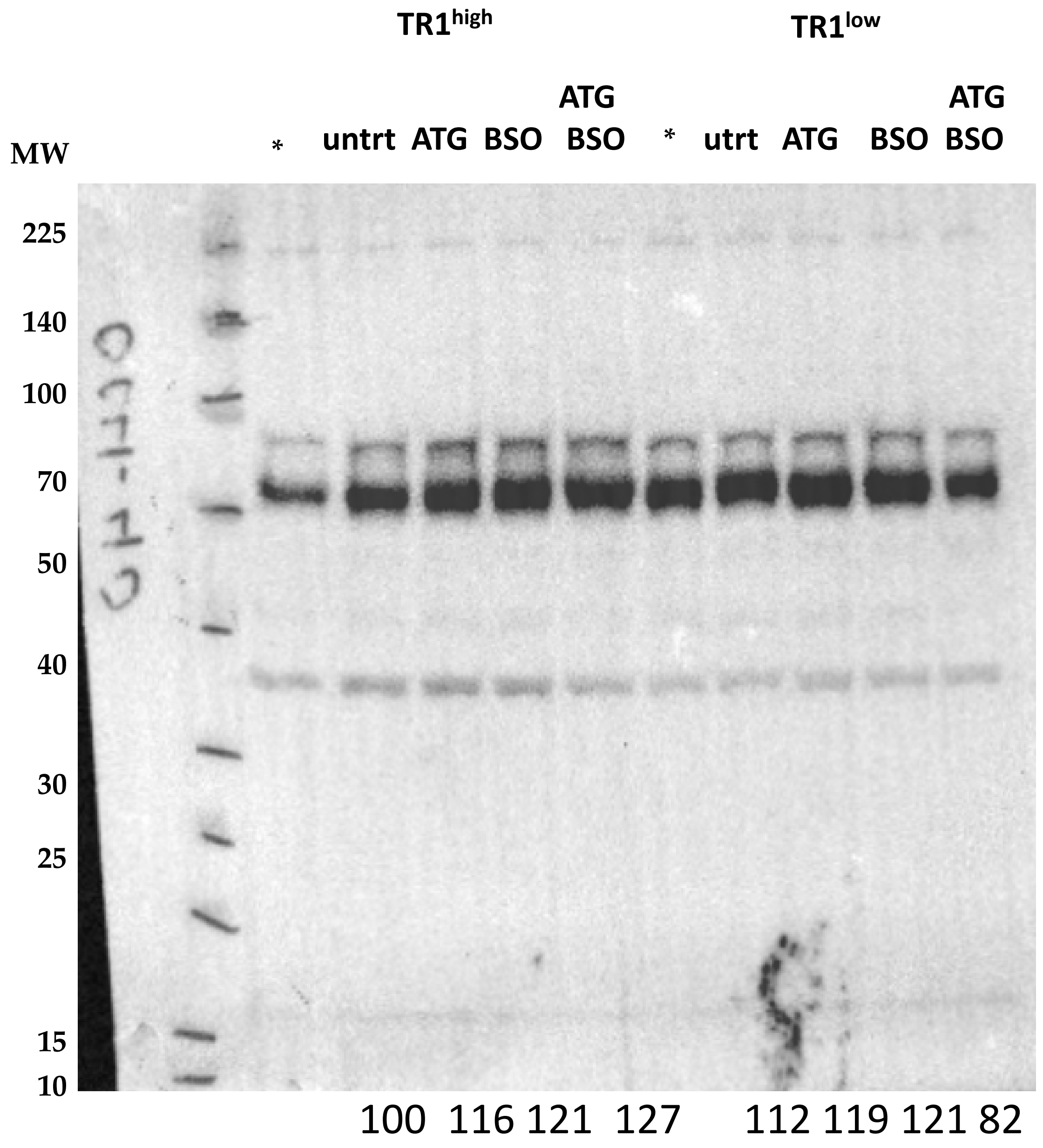

Figure 3b Western blot analysis comparing expression of TYRP1, TYR, and TR1 after 48-hour treatment with 10 μM BSO or 60 μM ATG or both in the TR1^{high} and TR1^{lo} cells. TR1 shown here. Note that positive control lanes (*) are from an unrelated experiment and do not appear in the figure.

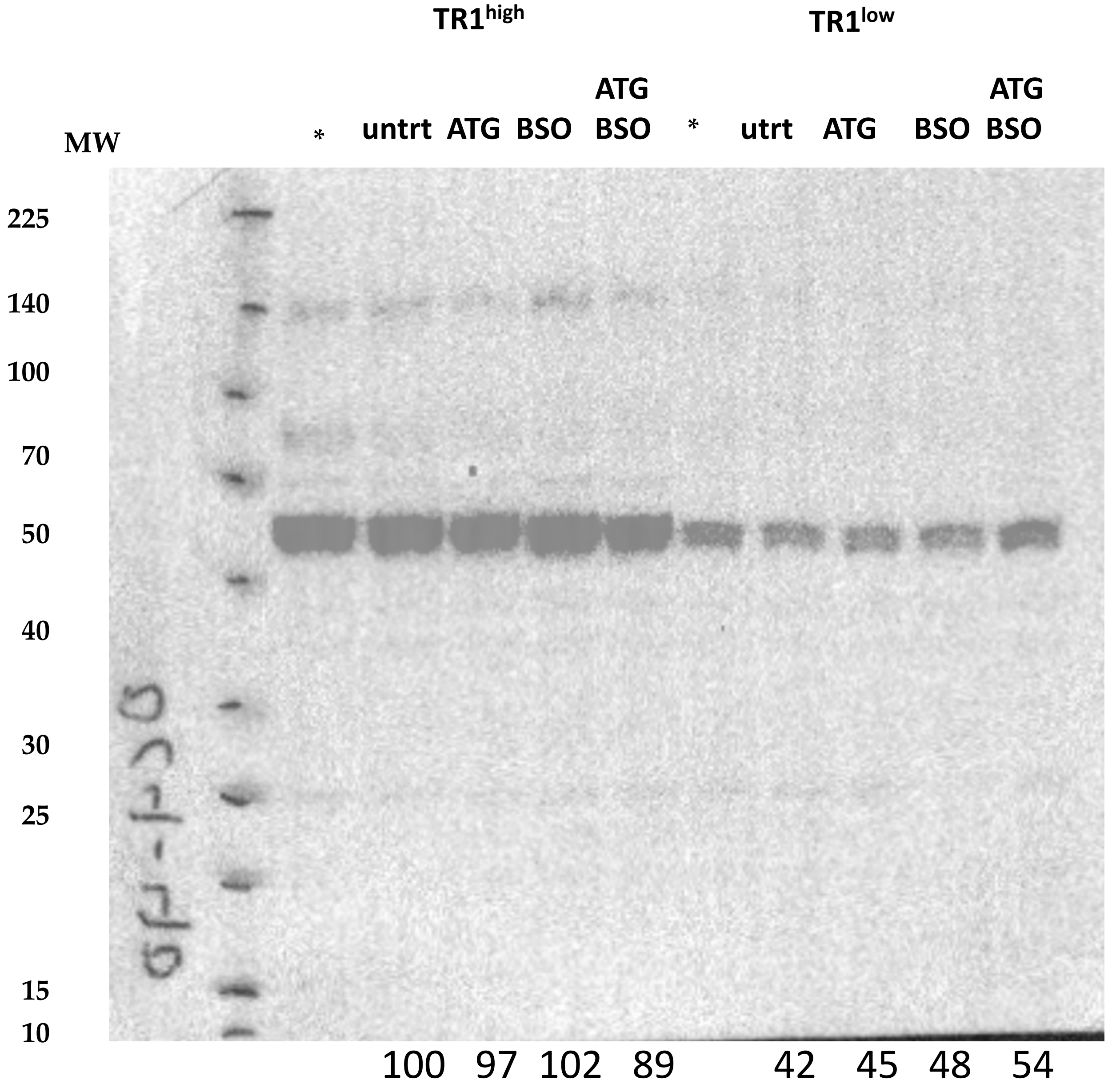

Figure 3b Western blot analysis comparing expression of TYRP1, TYR, MITF and TR1 after 48-hour treatment with 10 μM BSO or 60 μM ATG or both in the TR1[high] and TR1[lo] cells. MITF shown here. Note that positive control lanes (*) are from an unrelated experiment and do not appear in the figure.

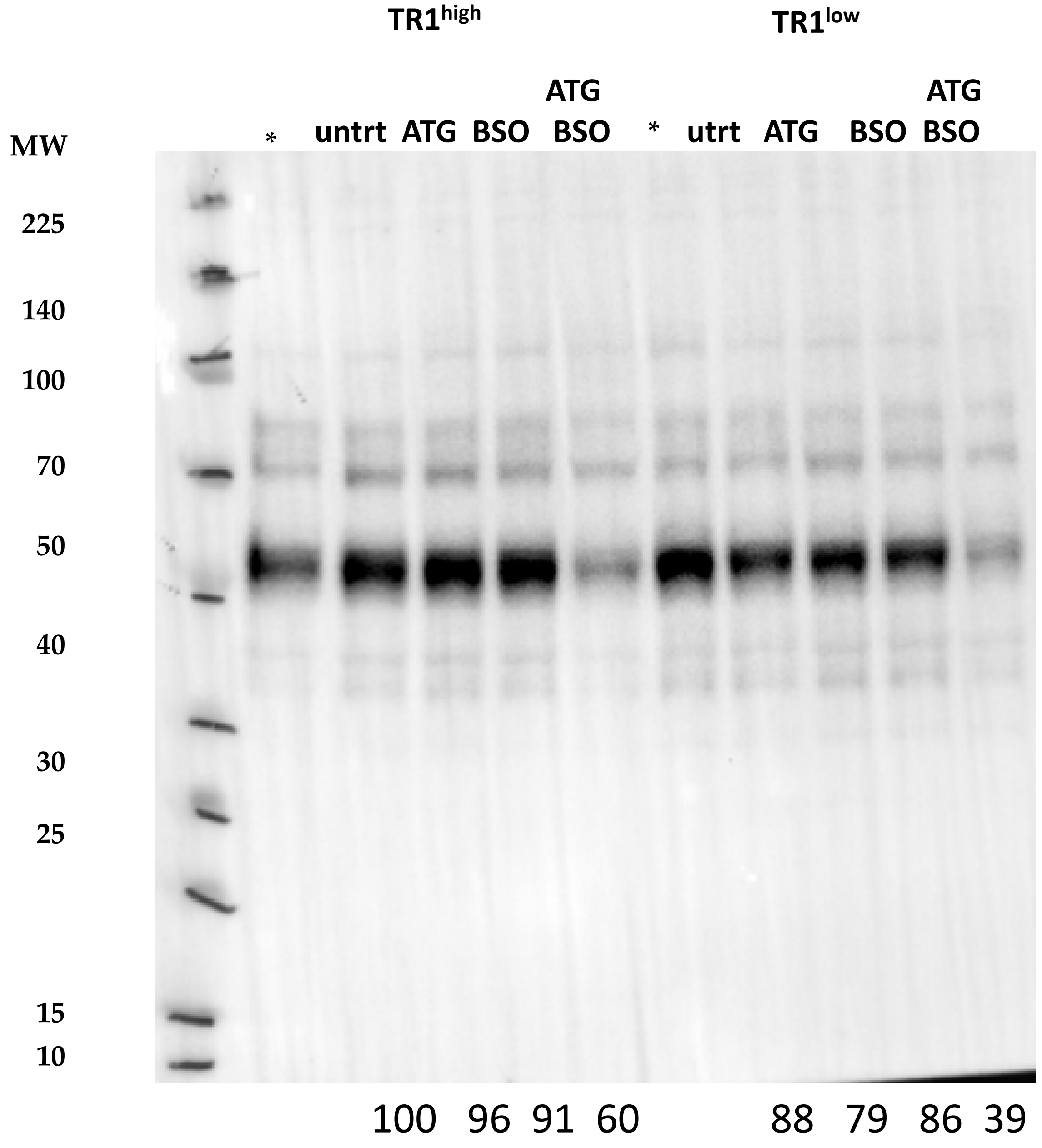

Figure 3b Western blot analysis comparing expression of TYRP1, TYR, MITF and TR1 after 48-hour treatment with 10 μM BSO or 60 μM ATG or both in the TR1^{high} and TR1^{lo} cells. Actin shown here. Note that positive control lanes (*) are from an unrelated experiment and do not appear in the figure.

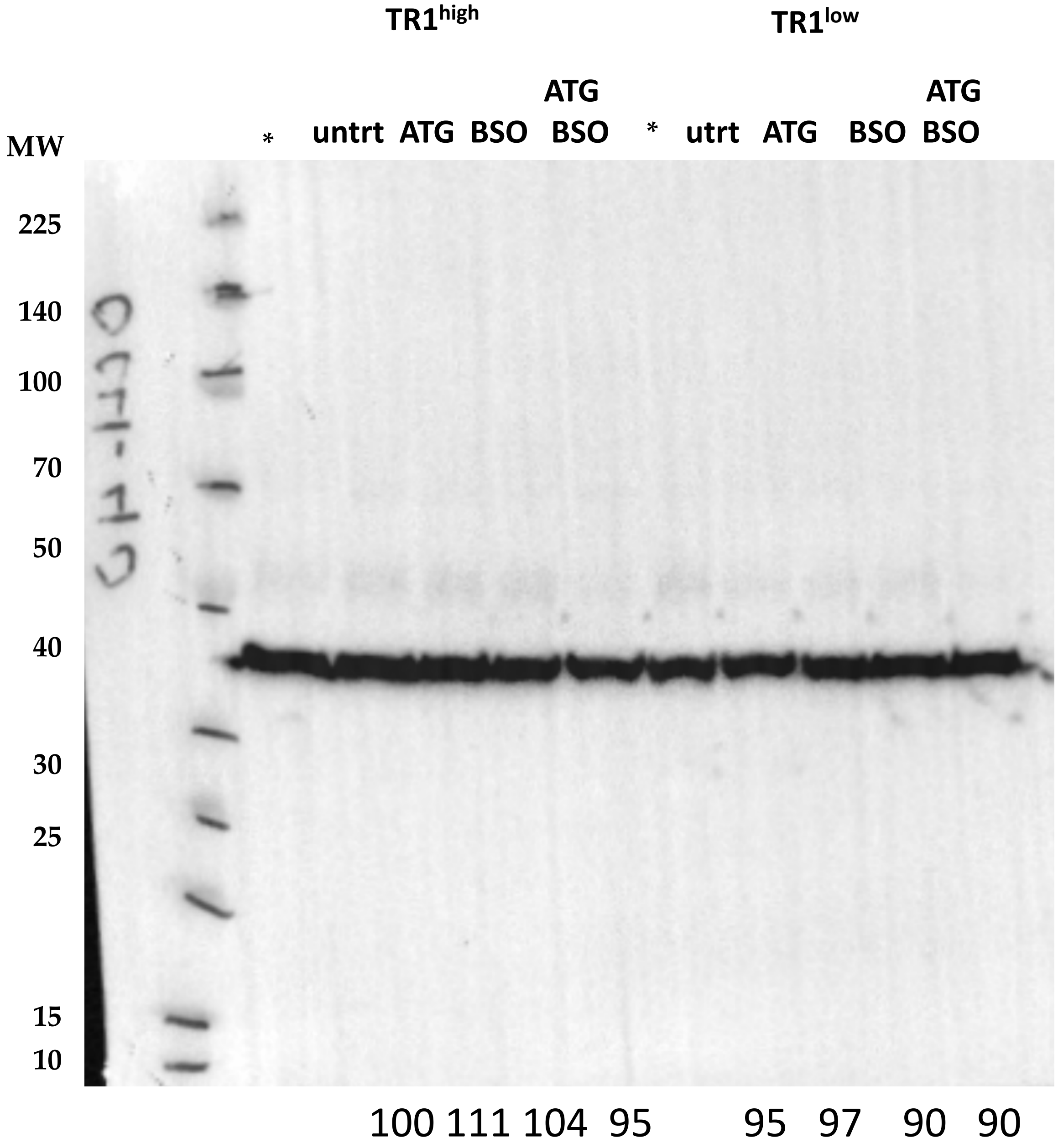

Figure 3c. Western blot analysis comparing protein expression of MITF, TYRP1, and TR1 in the TR1[high] and TR1[lo] cells after 24 and 48 hours of 5 μM PX12 treatment. Shown here is TYRP1

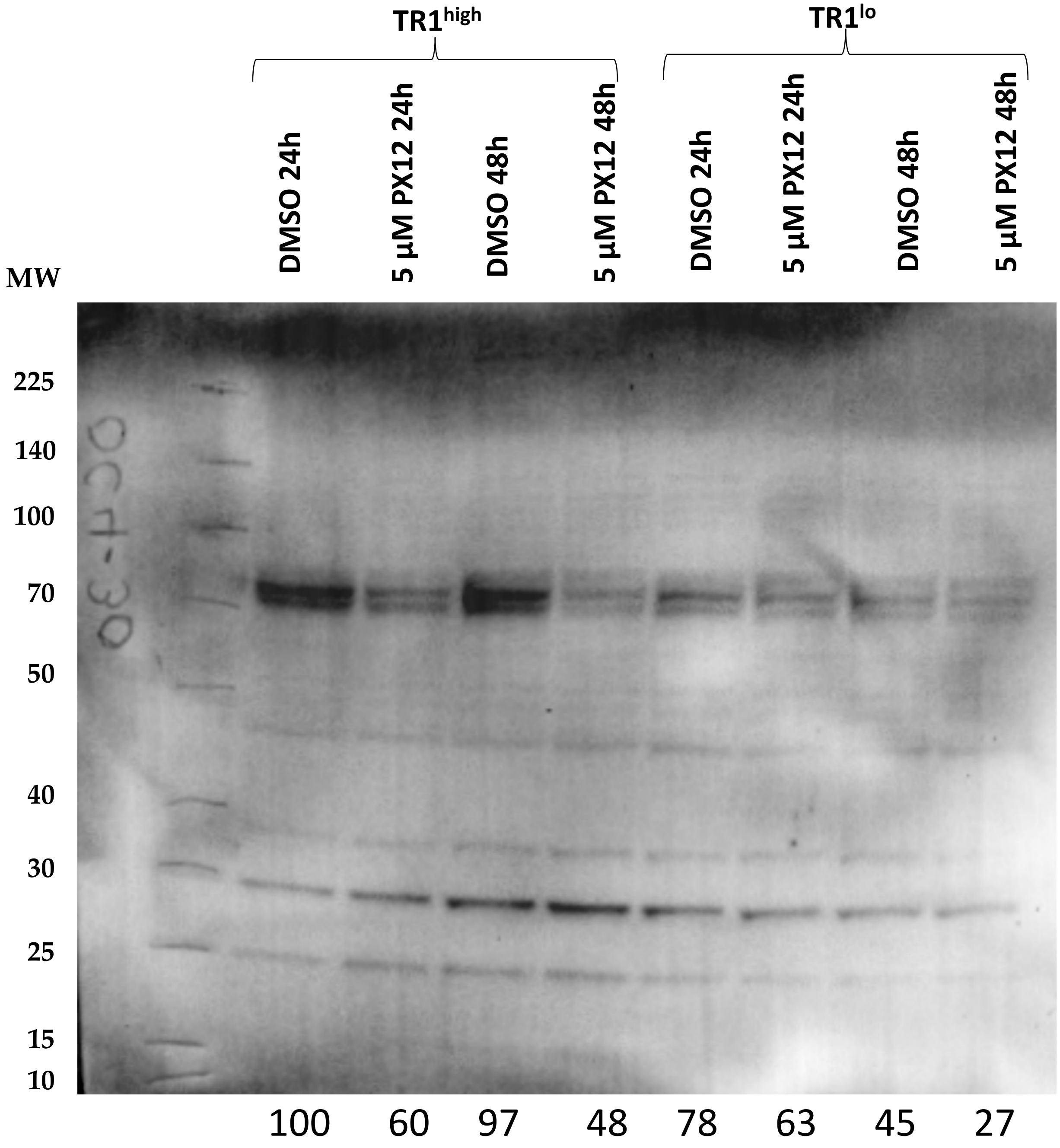

Figure 3c. Western blot analysis comparing protein expression of MITF, TYRP1, and TR1 in the TR1^{high} and TR1^{lo} cells after 24 and 48 hours of 5 µM PX12 treatment. Shown here is TYR

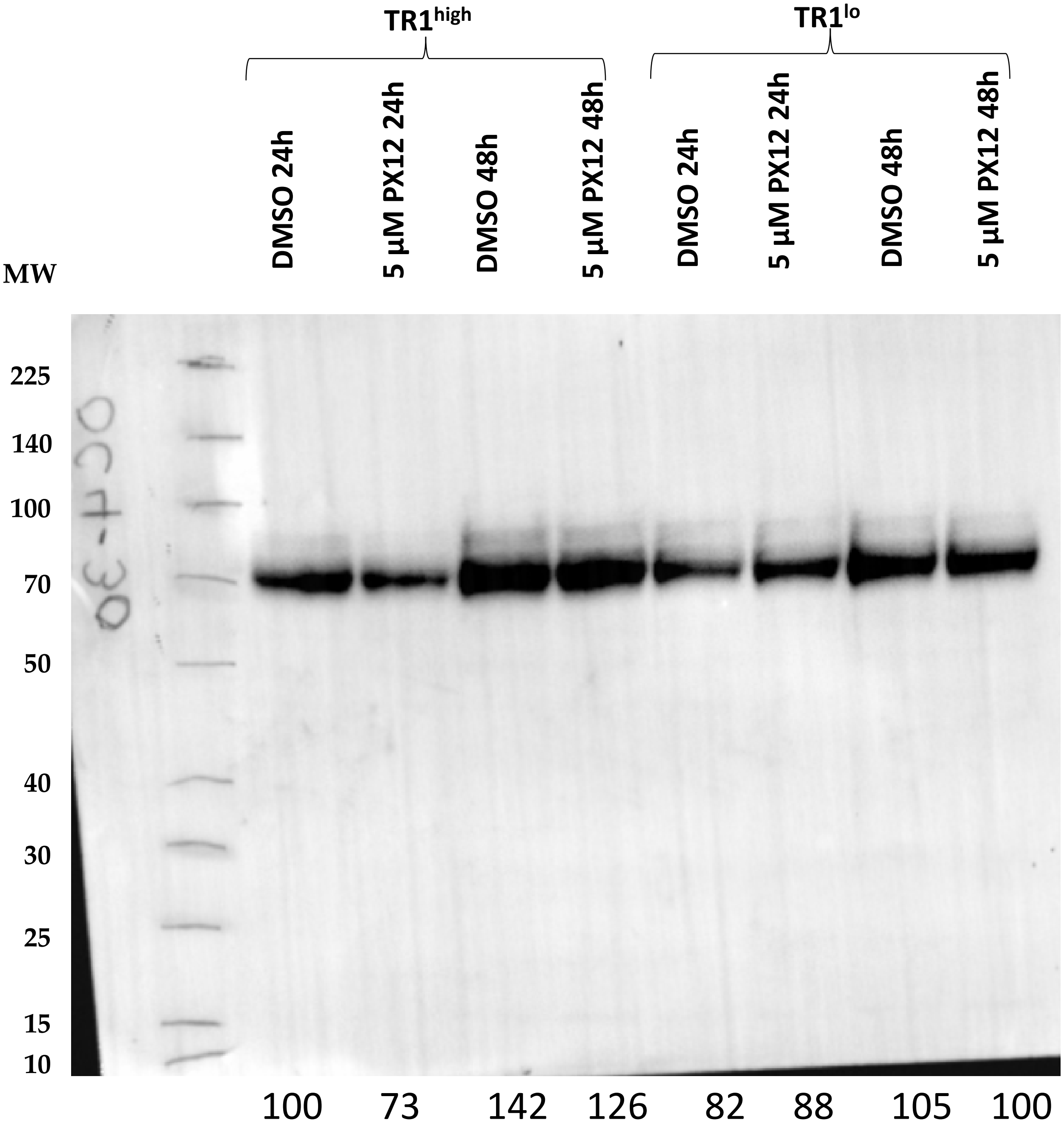

Figure 3c. Western blot analysis comparing protein expression of MITF, TYRP1, and TR1 in the TR1^{high} and TR1^{lo} cells after 24 and 48 hours of 5 μM PX12 treatment. Shown here is TR1

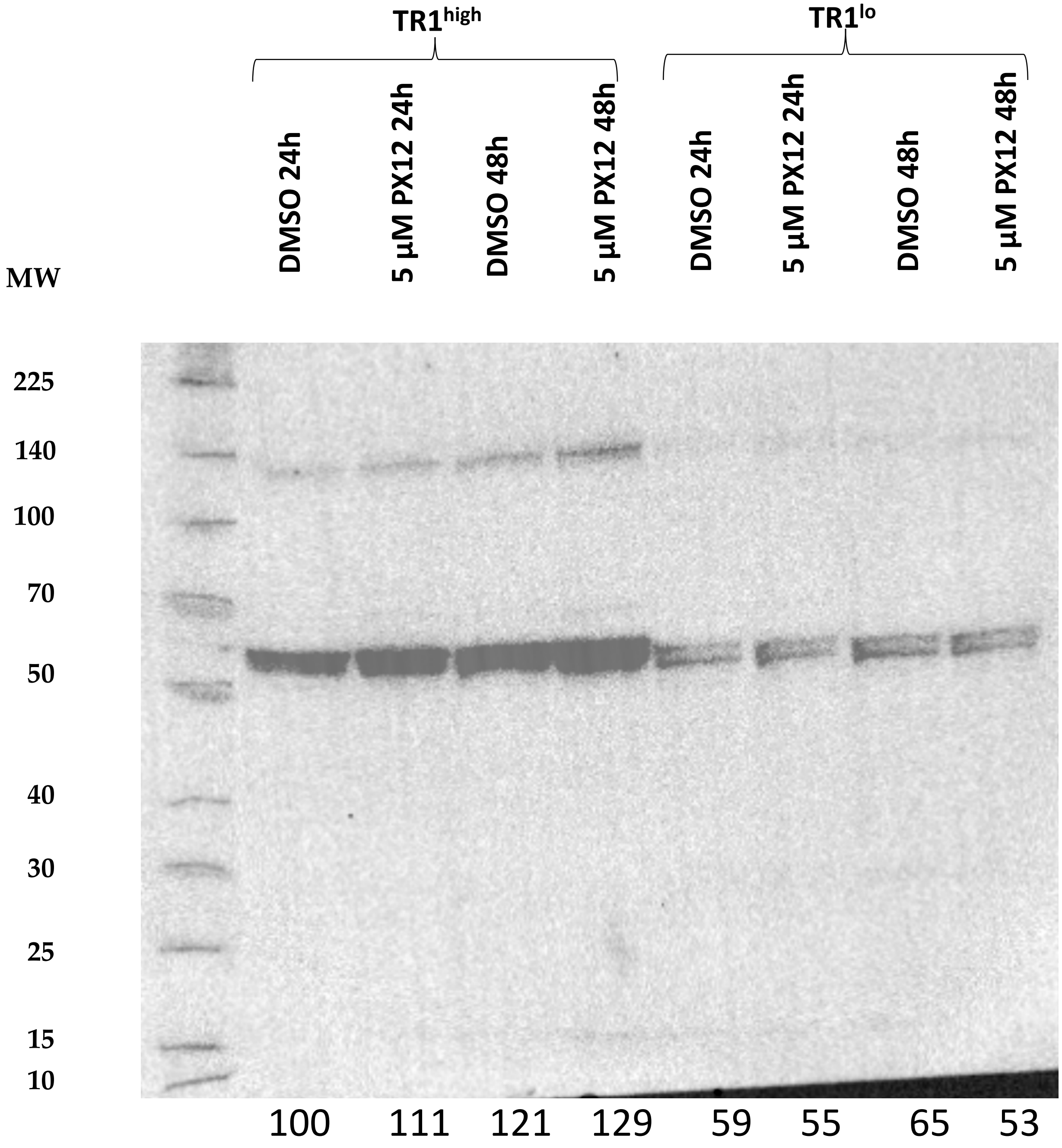

Figure 3c. Western blot analysis comparing protein expression of MITF, TYRP1, and TR1 in the TR1[high] and TR1[lo] cells after 24 and 48 hours of 5 µM PX12 treatment. Shown here is MITF

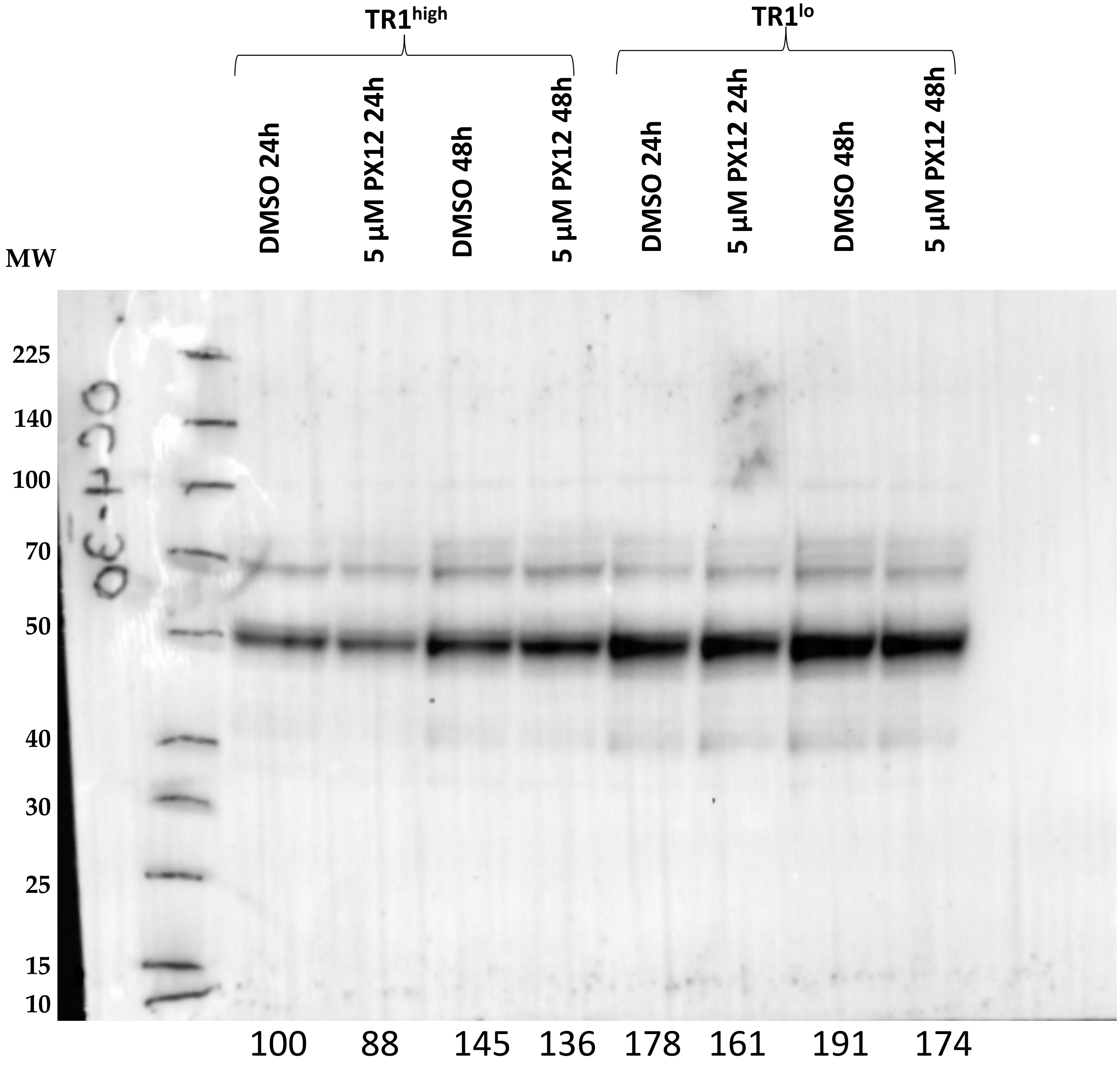

Figure 3c. Western blot analysis comparing protein expression of MITF, TYRP1, and TR1 in the TR1^high^ and TR1^lo^ cells after 24 and 48 hours of 5 µM PX12 treatment. Shown here is actin, which was imaged after TYR with no stripping

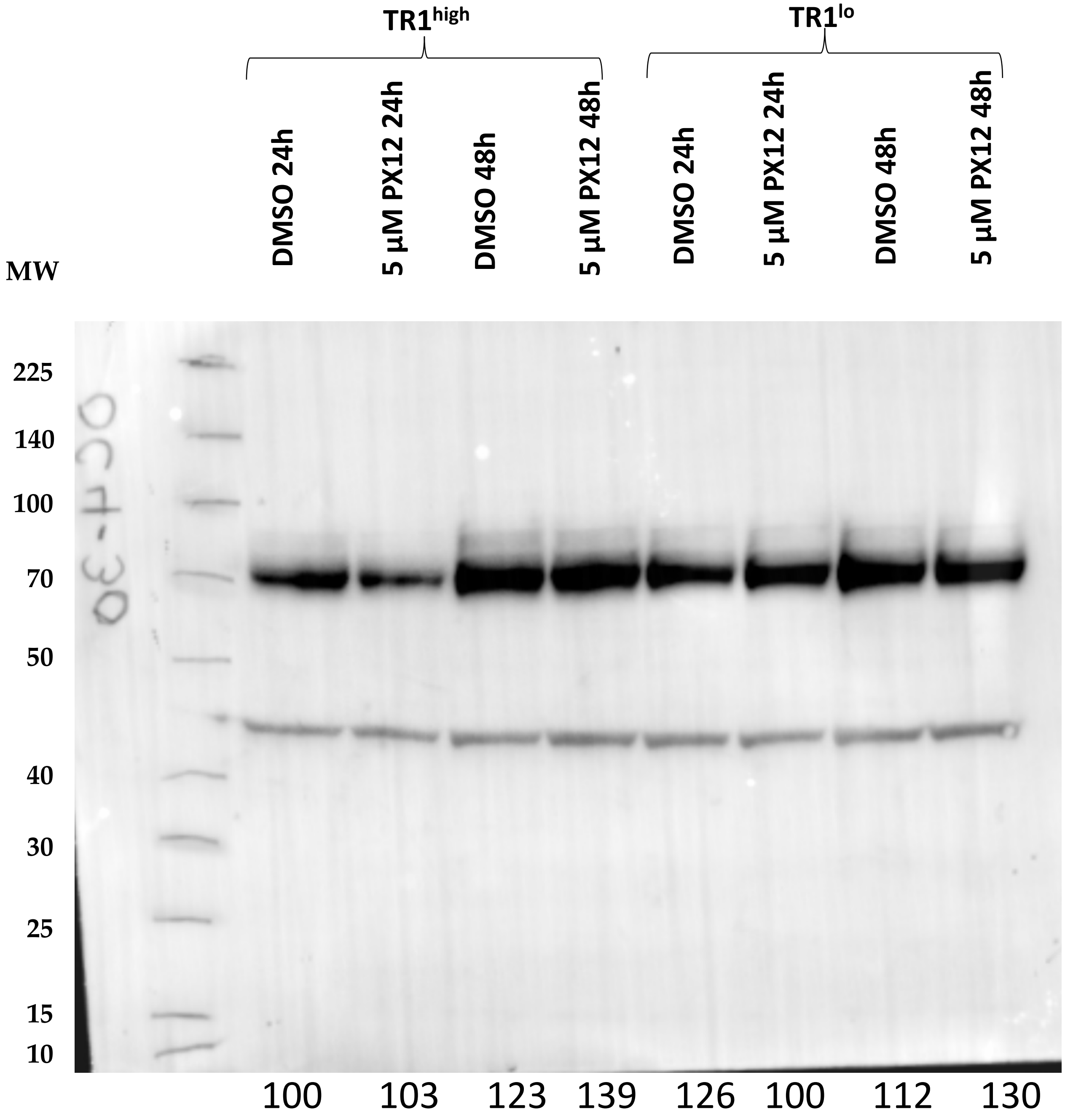

Figure 4a. Quantitation for these blots are as follows: **for PRX1, the three bands in untreated TR1^high cells are designated 100**; all other treatments in both cell lines are quantified relative to each of these three bands. The high-molecular weight (above 115 kD) PRX1 bands are not quantified. **For MITF, the untreated TR1^lo cells are designated 100**; all other treatments in both cell lines are quantified relative to this band. The exception is for the two high molecular weight bands. These are scanned from about 115 kD to the highest band above 225 kD for both 100 μM treatments. The bands in the TR1^lo cells are designated 100.

IB: anti-PRX1

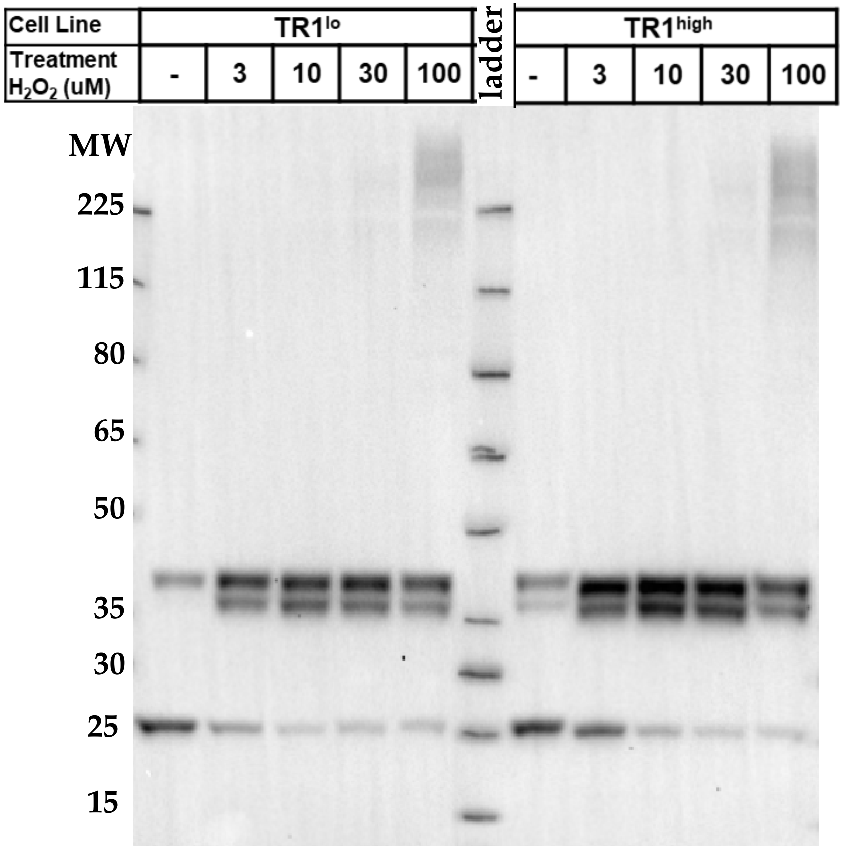

74	196	201	222	164	100	304	346	347	212
0	285	417	349	237	100	426	676	667	350
100	27	11	15	16	100	70	23	15	14

IB: anti-MITF

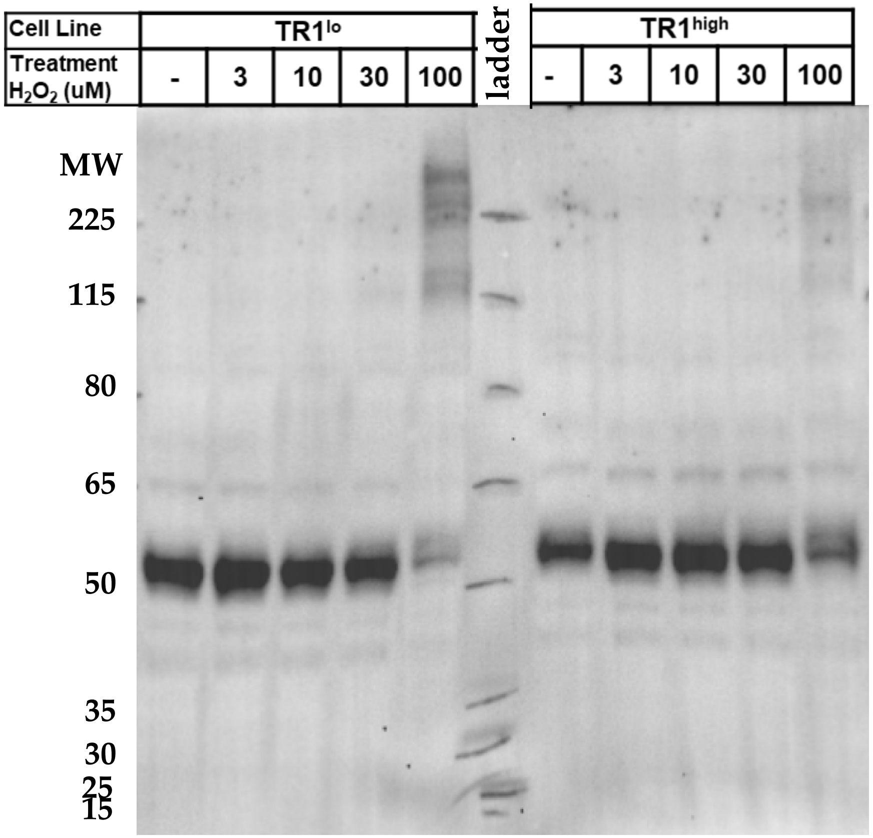

100	110	81	77	20	63	99	93	82	59
				100					61

Figure 4a. β-actin blots - The quantification for these blots is calculated relative to the untreated TR1[lo] cells, which are set to 100.

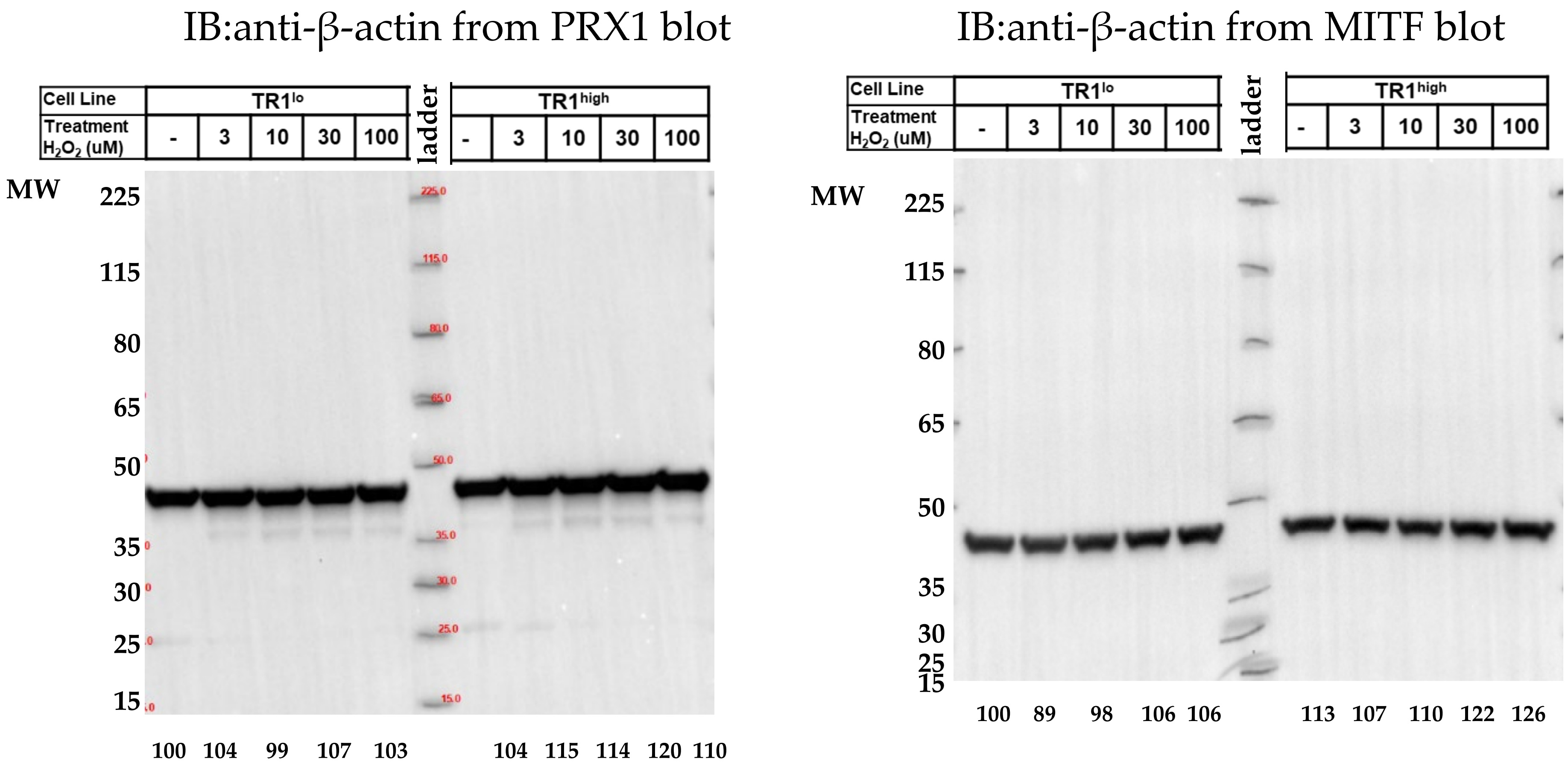

Figure 4b. Reducing gels. TR1[lo] untreated lane on each blot is set to 100.

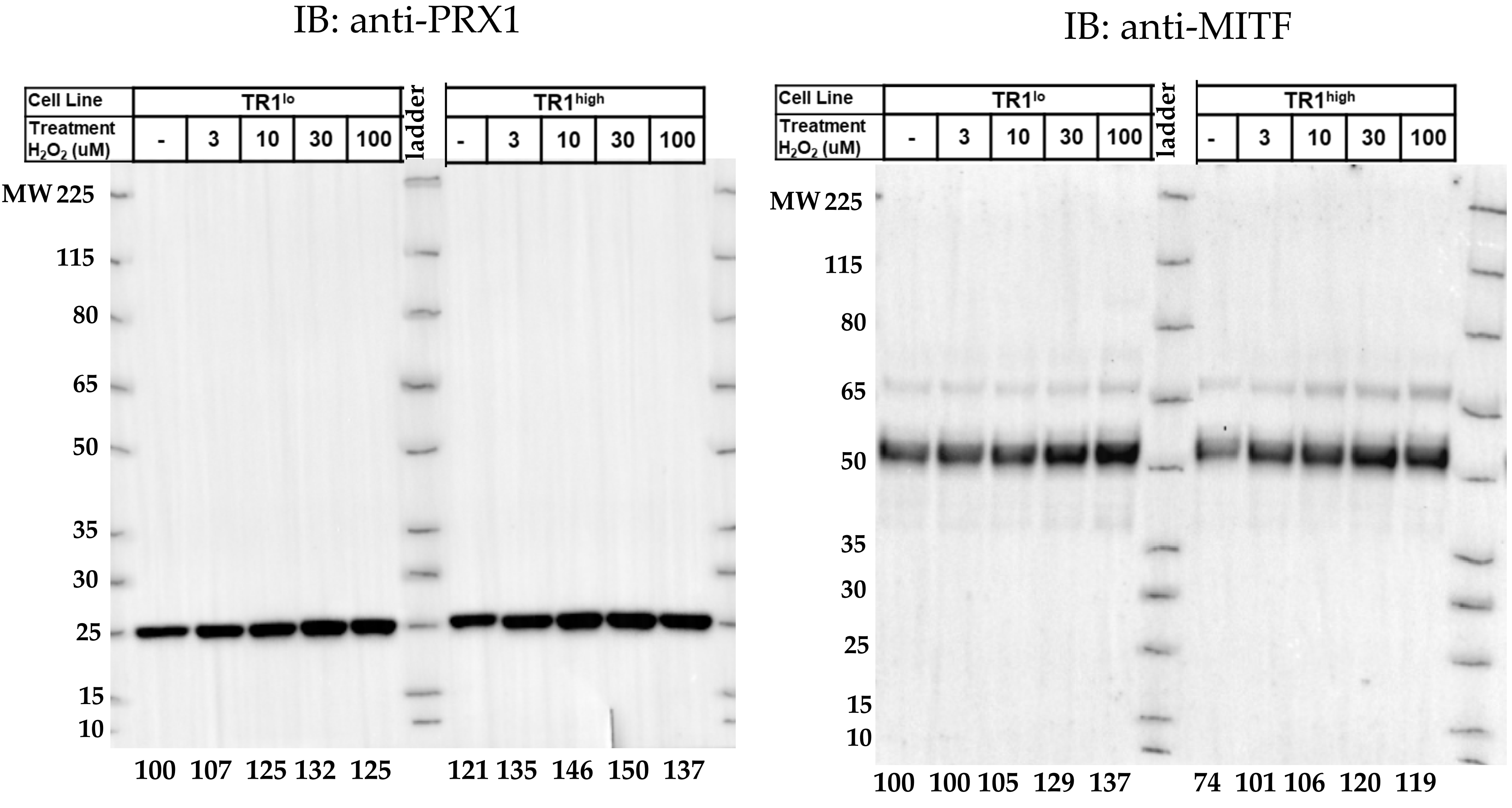

Figure 4b. Reducing gels. TR1[lo] untreated lane on each blot is set to 100.

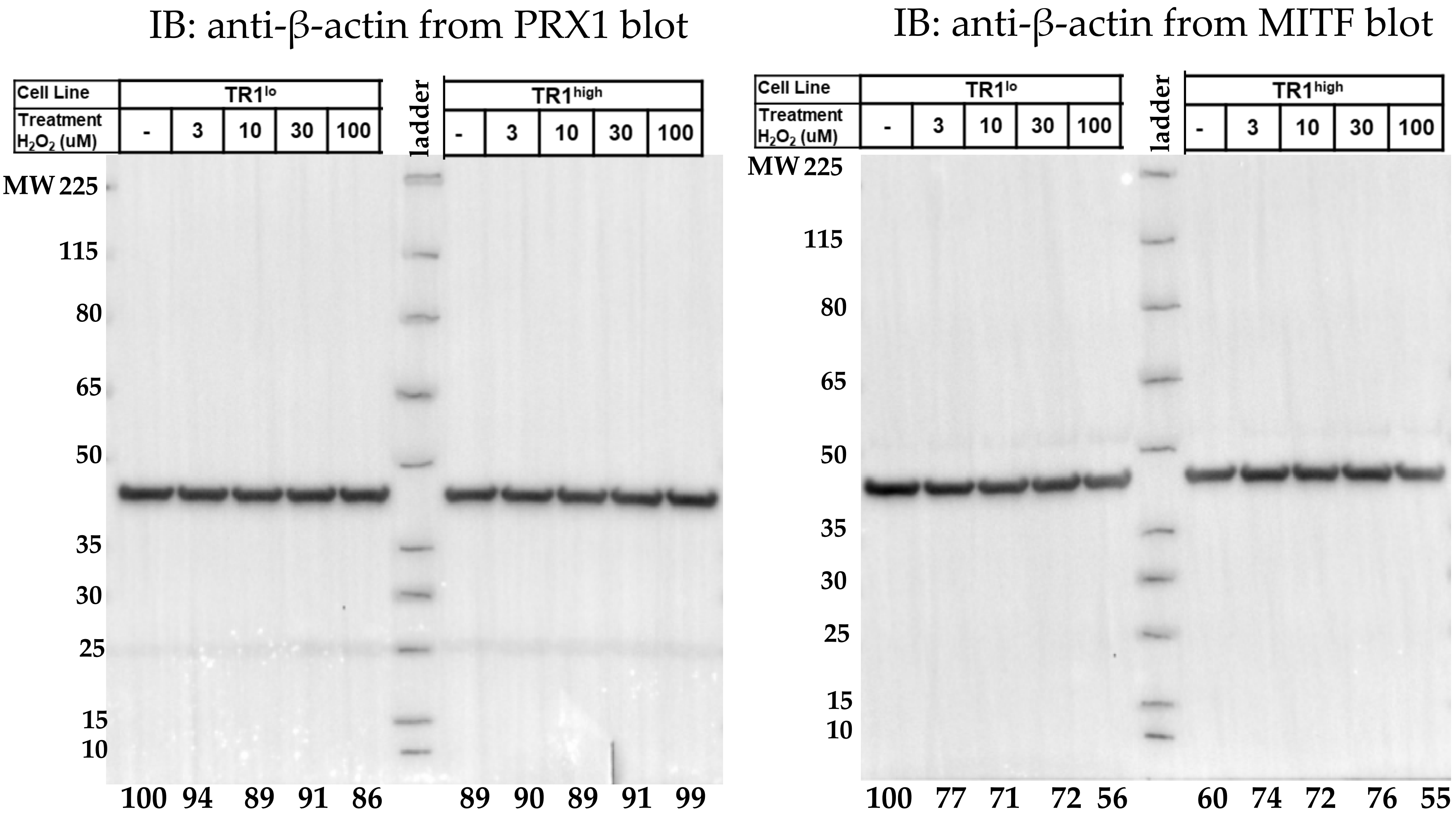

Figure 6. Non-Reducing gels: The no H_2O_2 lane for the PIG1 cell line was set to 100 for quantitation of the bands at 50 kD (for MITF) and 40 kD (for β-actin). The 100 μM H_2O_2 lane for the PIG1 cells was set to 100 for the above 115 kD band for MITF.

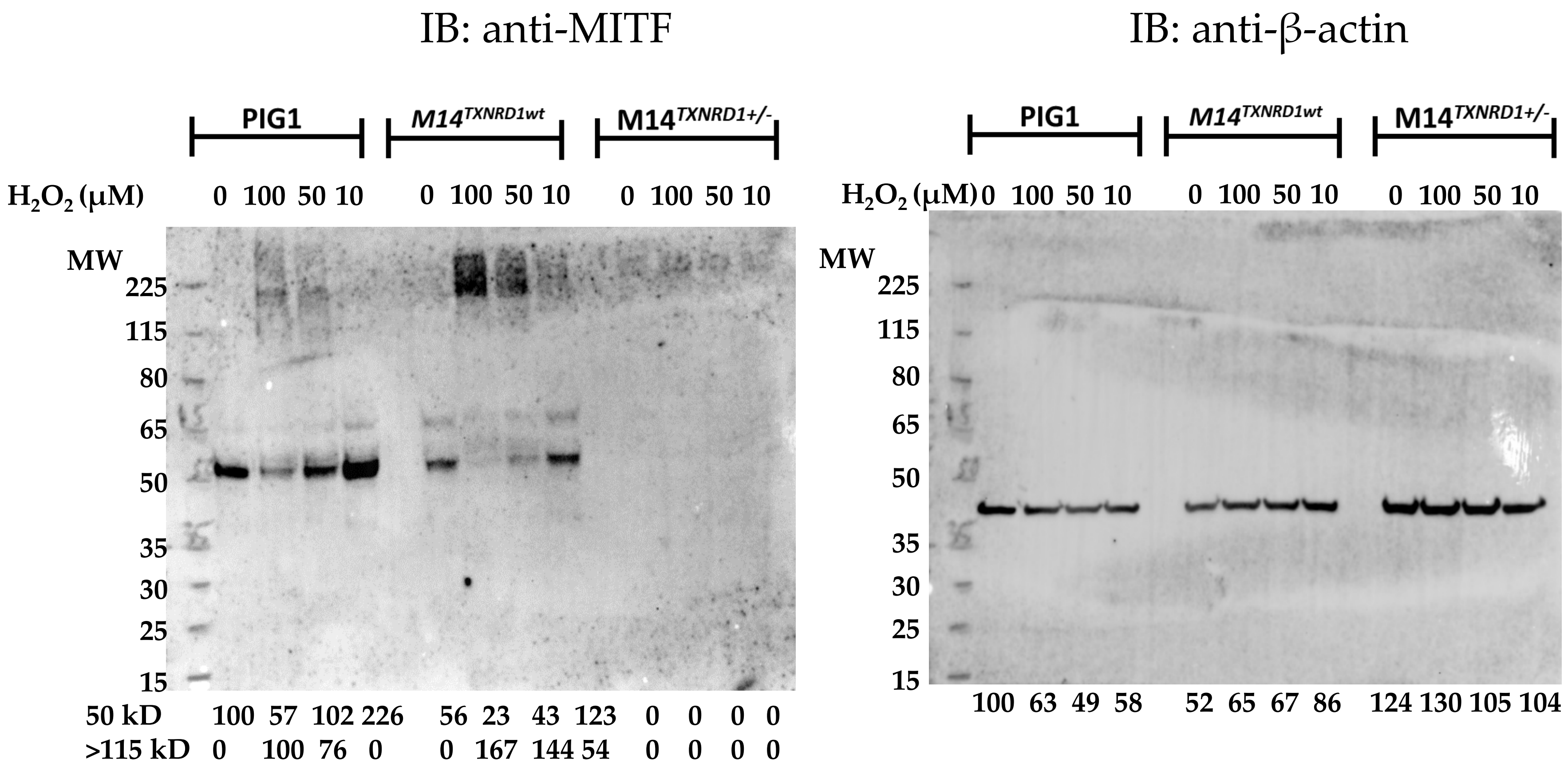

Figure 6. Reducing gels: The no H_2O_2 lane for the PIG1 cell line was set to 100 for quantitation of the bands of both MITF and β-actin.

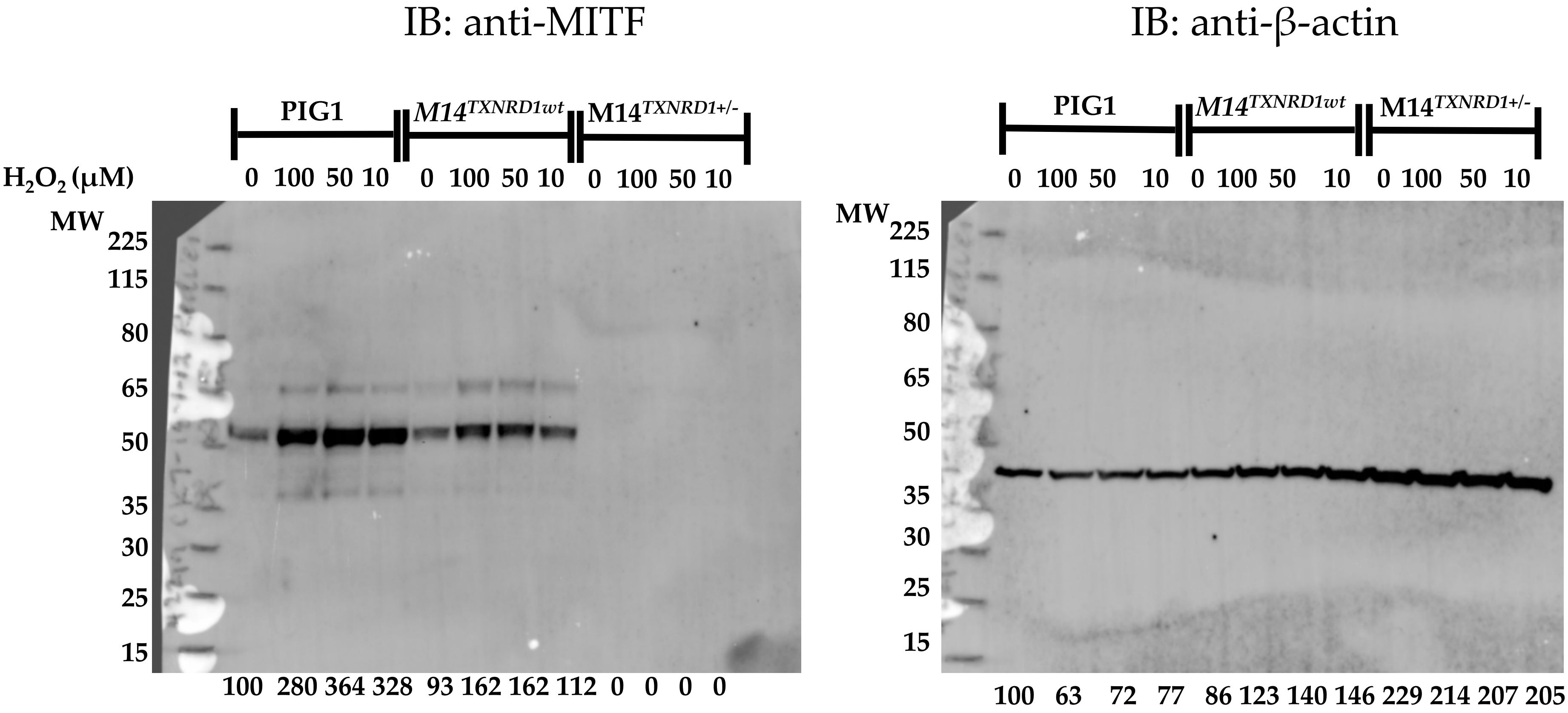

Supplement: Supplementary file 1 [file cancers-14-05011-s001.zip › cancers-1836209 WB.pdf]
