# Supplementary material for: MITF Is Regulated by Redox Signals Controlled by the Selenoprotein Thioredoxin Reductase 1
Source: Cancers (Basel). 2022 Oct 13;14(20):5011. doi: 10.3390/cancers14205011 (PMC9600194; doi:10.3390/cancers14205011)
Supplement: Supplementary file 1 [file cancers-14-05011-s001.zip › cancers-1836209-Figures and Tables.pdf]

SUPPLEMENTAL FIGURES

Supplementary Figure S1.

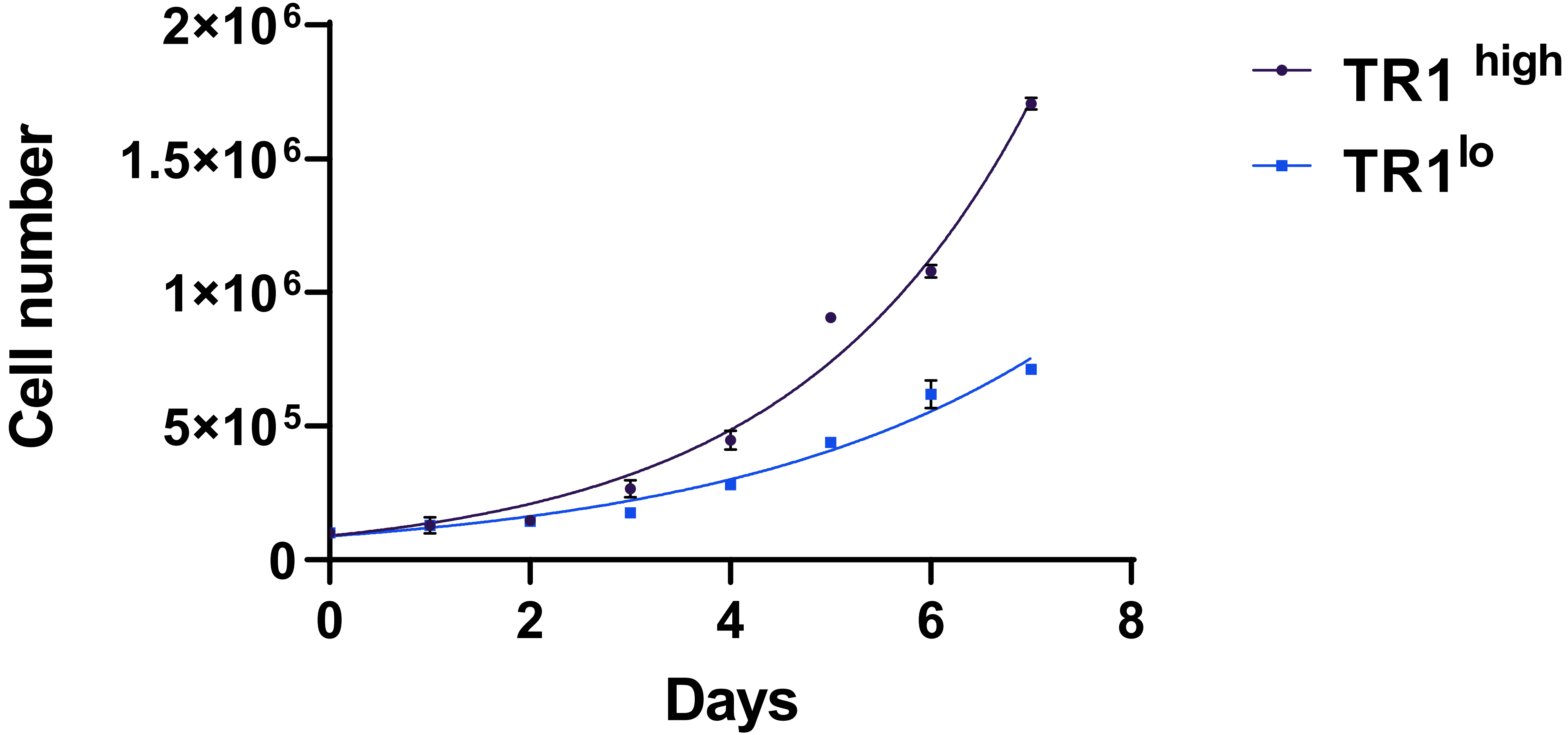

	TR1high	95%CI	TR1lo	95%CI
Doubling Time (days)	1.65	1.48-1.83	2.26	2.01-2.57

95%CI= 95% confidence interval

Supplementary Figure S2. Knockdown of TR1 using siRNAs directed against TR1 with three different transfection reagents results in decreases in TYRP1 and TYR proteins. M14 melanoma cells were treated with siRNAs targeting human *TXNRD1* (Dharmacon siRNA SMARTpool cat # L-008236-00-0010) or non-targeting control siRNAs (cat# D-001810-01-05) using three different transfection reagents. After 48 hours cells were harvested and lysates were analyzed by Western blot.

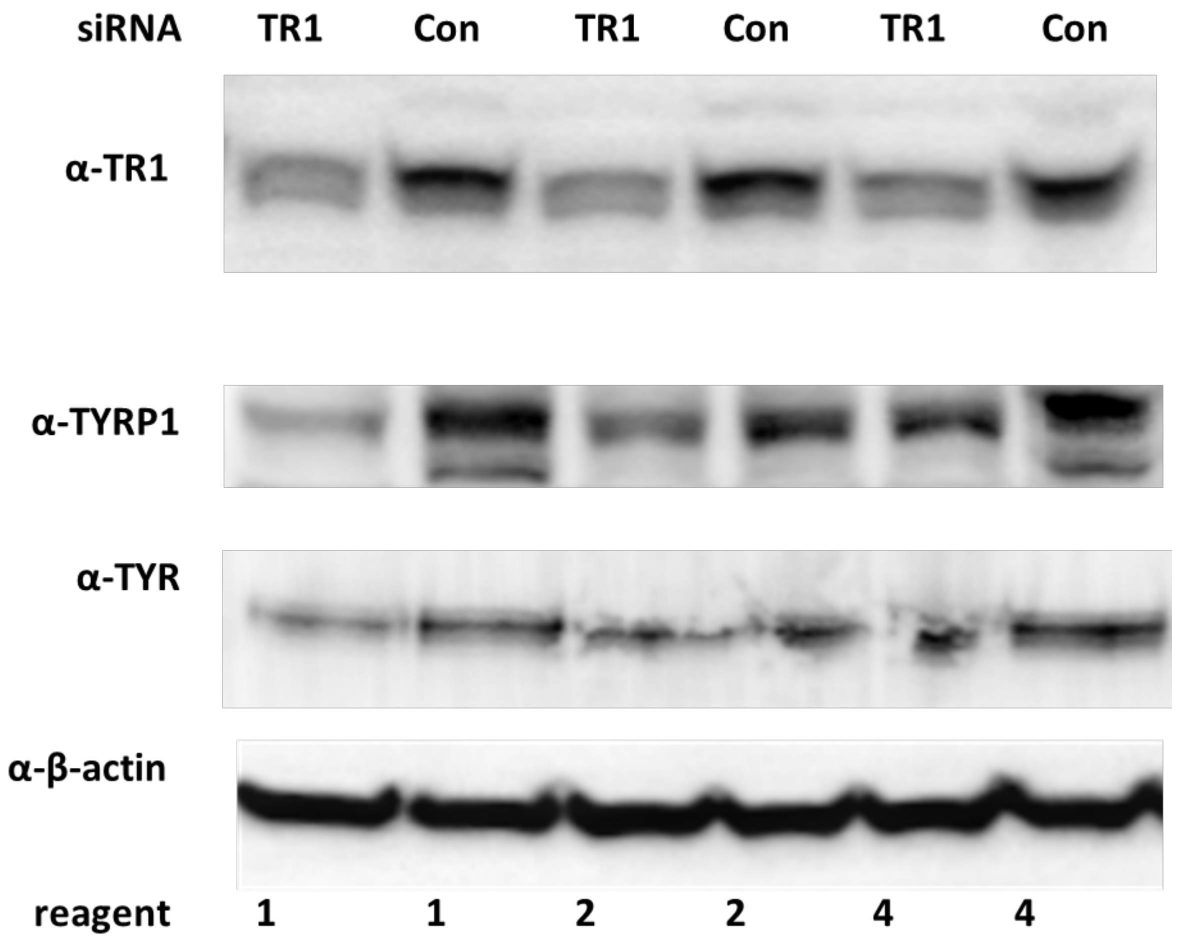

Supplementary Figure S3.

	TR1 expression log2 Fold Change	p value*
TYR	-8.71	5.59E-09
TYRP1	-3.60	2.88E-06
PMEL	-4.09	6.61E-06
MLANA	-4.35	1.70E-07
DCT	-7.48	2.33E-06

RNAseq data for M14$^{TXNRD1+/-}$ B

*(adjusted for multiple comparisons)

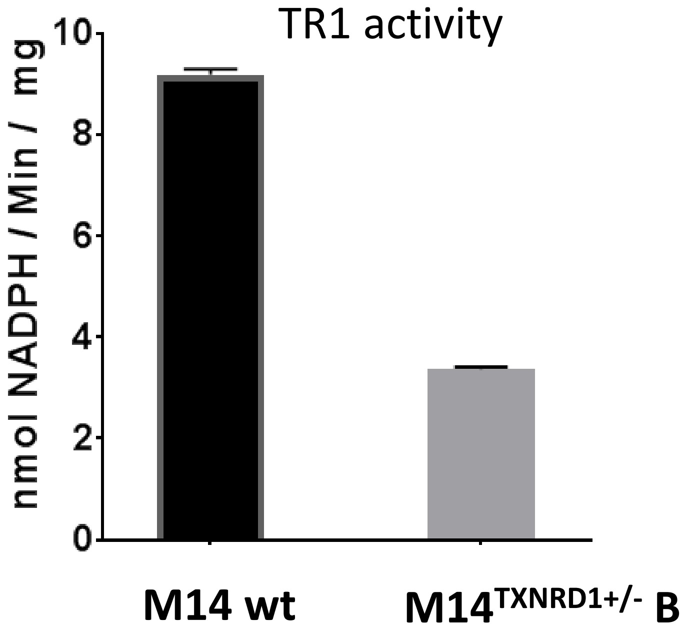

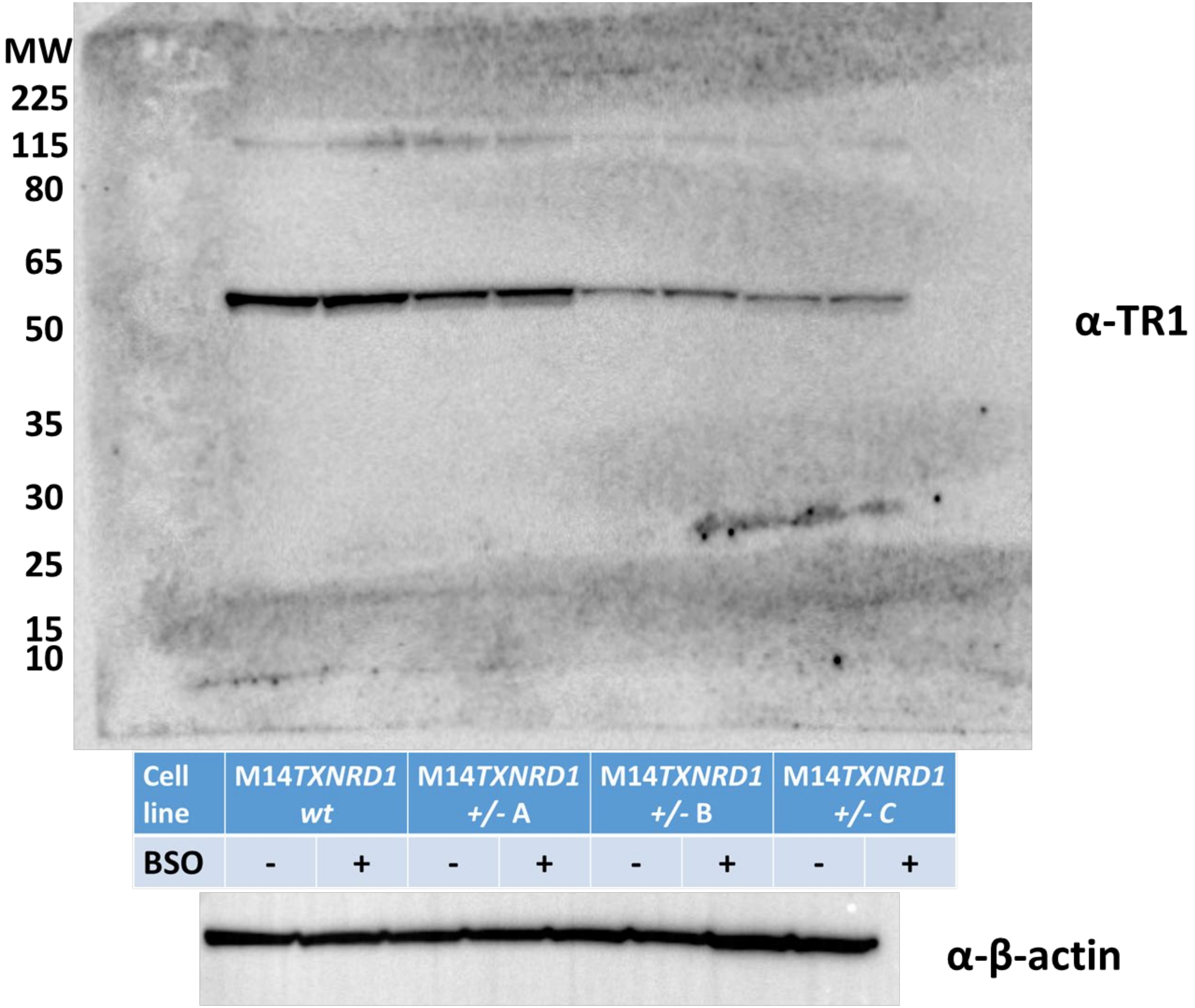

Supplementary Figure S4.

Mutations in M14$^{TXNRD1+/-}$ B cell line

	Sequence (Exon 9), **BOLD**= Cas9 Target, ***TGG*****=** PAM site, **Mutant sequence**, *= stop codon
Exon 9 wt sequence	GATGATCTTTTCTCCTTGCCTTACTGCCCGGGTAAGACCCTGGTTGTT**GGAGCATCCTATGTCGCTTTGGAGT** GCGCTGGATTTCTTGCTGGTATTGGTTTAGACGTCACTGTTATGGTTAGGTCCATTCTTCTTAGAGGATTTGAC CAGGACATGGCCAACAAAATTGGTGAACACATGGAAGAACATGGCATCAAGTTTATAAGACAGTTCGTACCA ATTAAA
Amino acid sequence	DDLFSLPYCPGKTLVV**GASYVALE**CAGFLAGIGLDVTVMVRSILLRGFDQDMANKIGEHMEEHGIKFIRQFVPIK
**CT DEL, NT:**	GATGATCTTTTCTCCTTGCCTTACTGCCCGGGTAAGACCCTGGTTGTTGGAGCATCCTATGTCG__TTGGAGTG CGCTGGATTTCTTGCTGGTATTGGTTTAGACGTCACTGTTATGGTTAGGTCCATTCTTCTTAGAGGATTTGACC AGGACATGGCCAACAAAATTGGTGAACACATGGAAGAACATGGCATCAAGTTTATAAGACAGTTCGTACCAA TTAAA
**AA :**	DDLFSLPYCPGKTLVV**GASYV**VGVRWISCWYWFRRHCYG*VHSS*RI*PGHGQQNW*THGRTWQVYKTVRTN
**TCGCT DEL, NT:**	GATGATCTTTTCTCCTTGCCTTACTGCCCGGGTAAGACCCTGGTTGTTGGAGCATCCTATG______TTGGAGTG CGCTGGATTTCTTGCTGGTATTGGTTTAGACGTCACTGTTATGGTTAGGTCCATTCTTCTTAGAGGATTTGACC AGGACATGGCCAACAAAATTGGTGAACACATGGAAGAACATGGCATCAAGTTTATAAGACAGTTCGTACCAA TTAAA
**AA :**	DDLFSLPYCPGKTLVV**GASYV**GVRWISCWYWFRRHCYG*VHSS*RI*PGHGQQNW*THGRTWHQVYKTVRTN*

Supplementary Figure S5

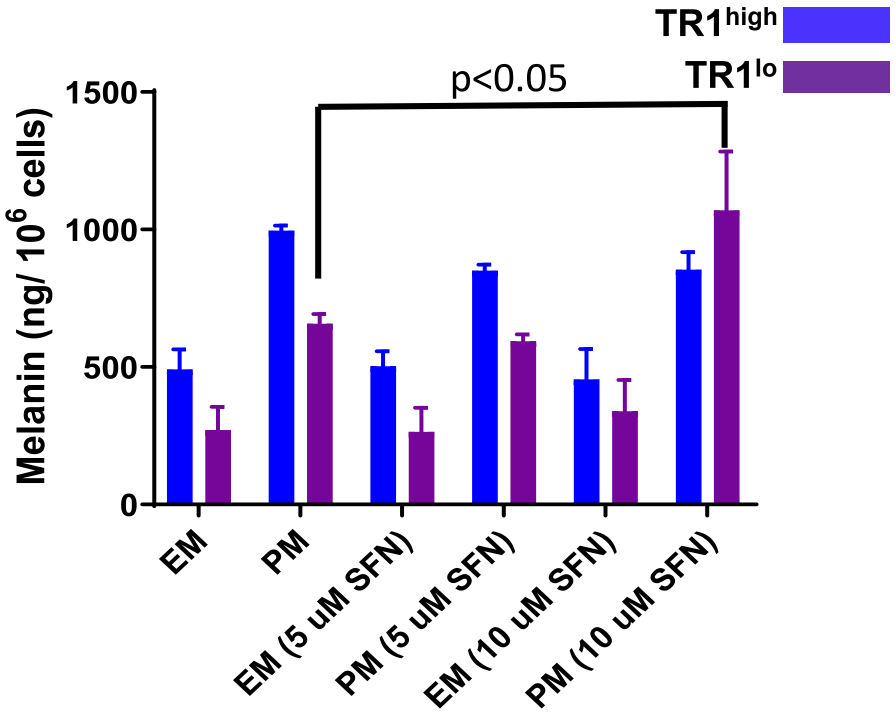

Supplementary Figure S6. qPCR analysis of TR1^{lo} cells relative to TR1^{high} .

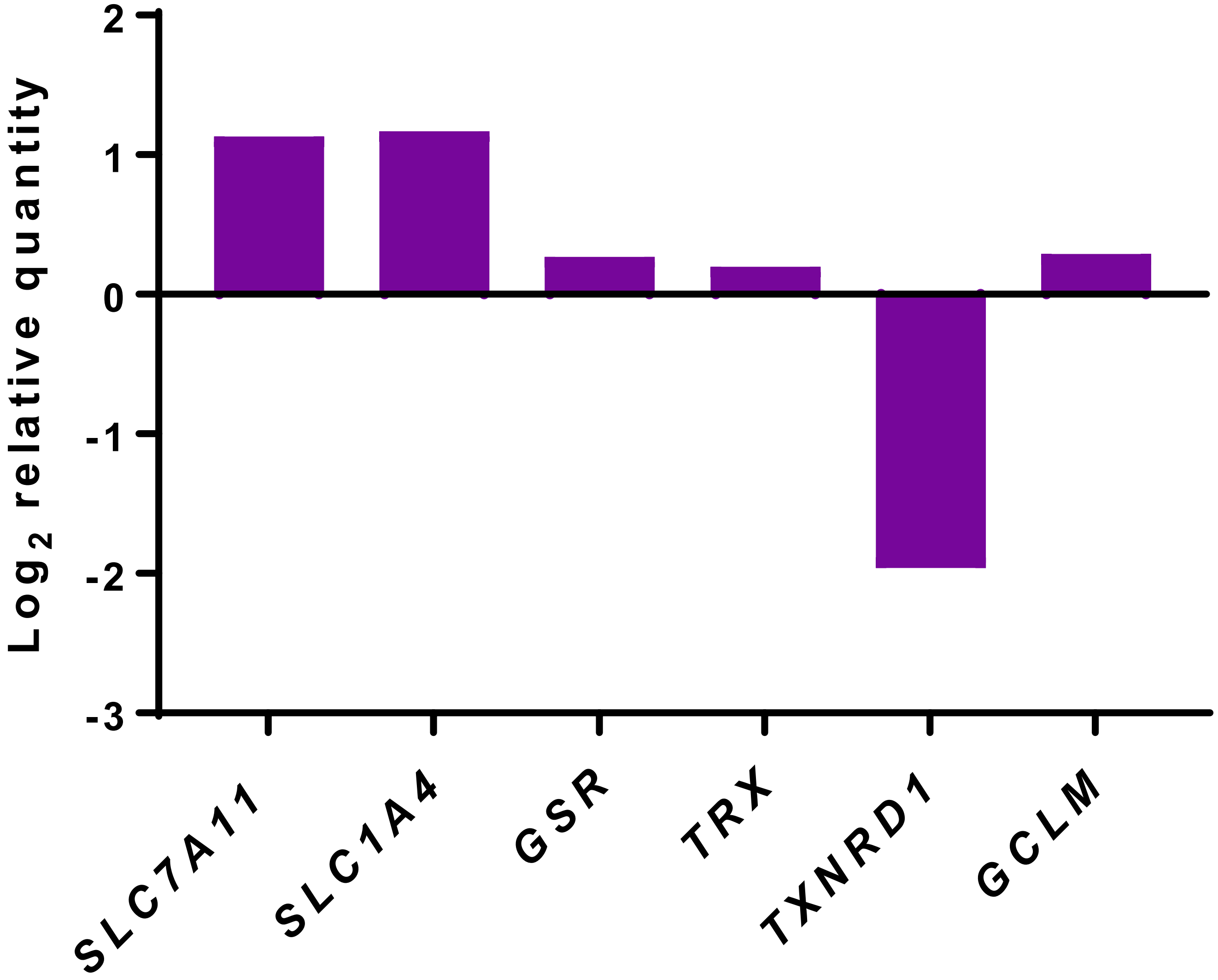

Supplementary Figure S7.

a

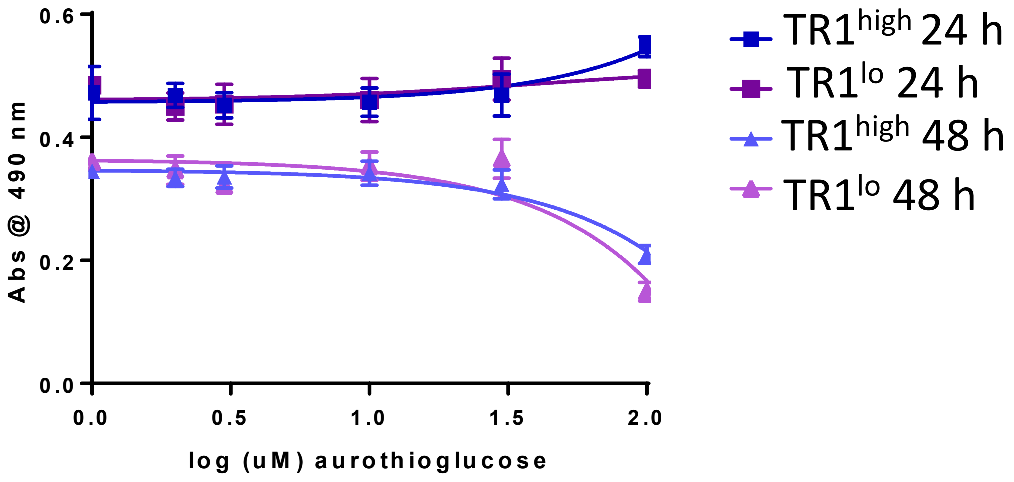

b

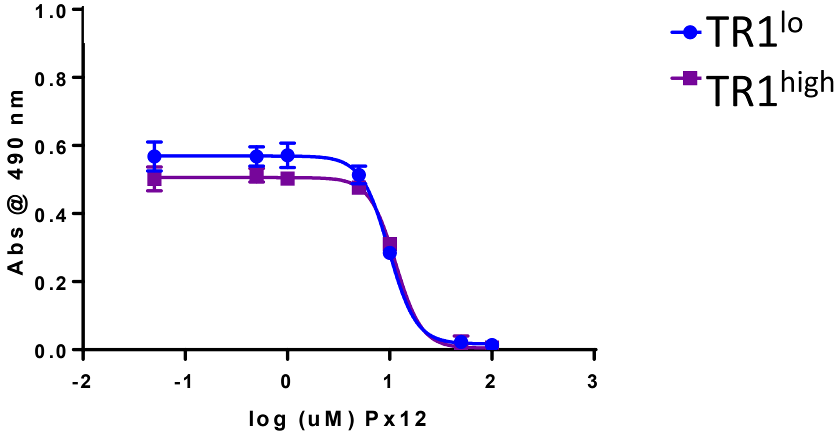

Supplementary Figure S8.

Figure 3B
serum Lot A

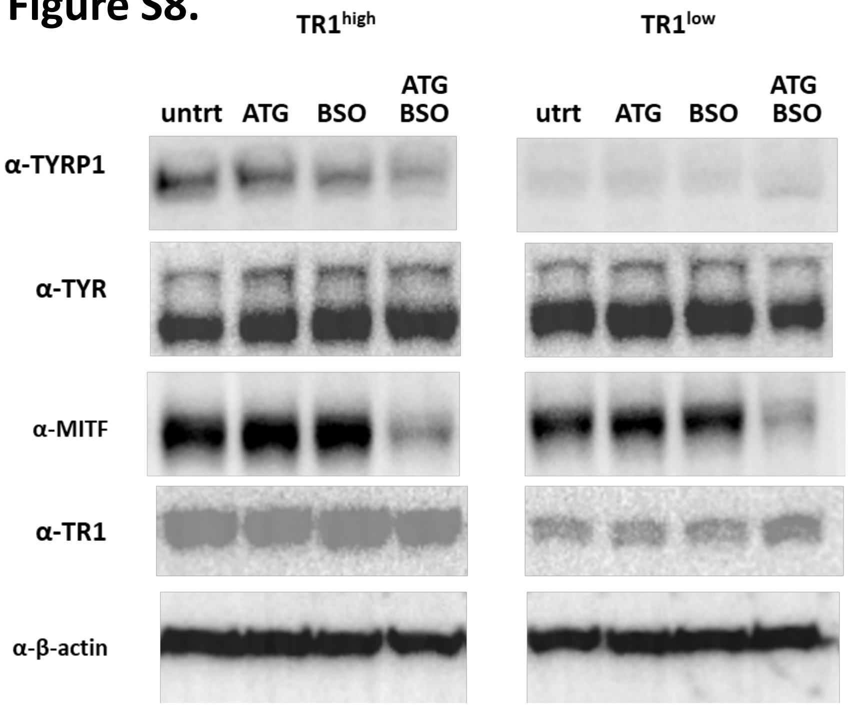

Figure 3C
serum Lot A

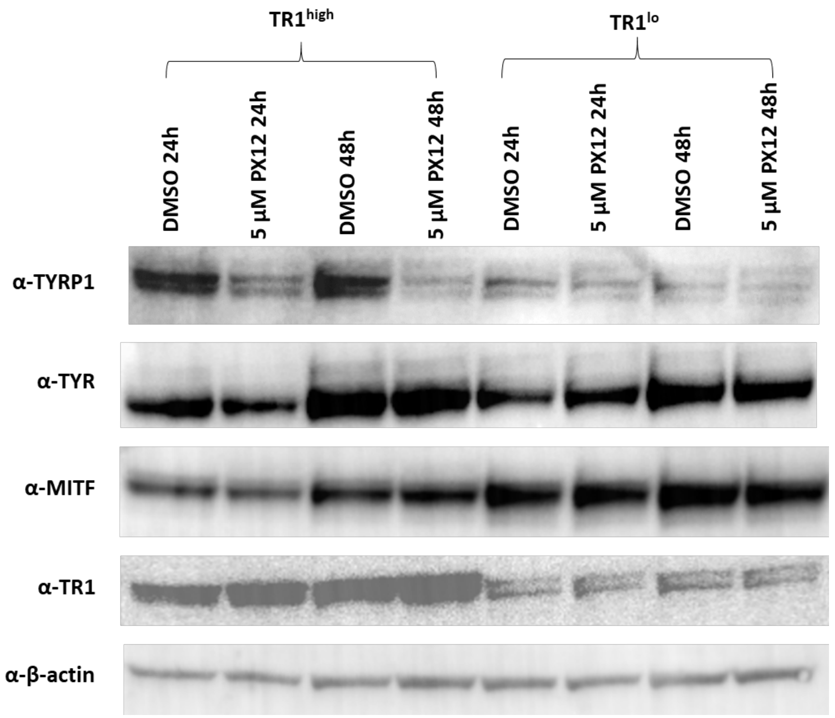

Previous experiment
Serum Lot B

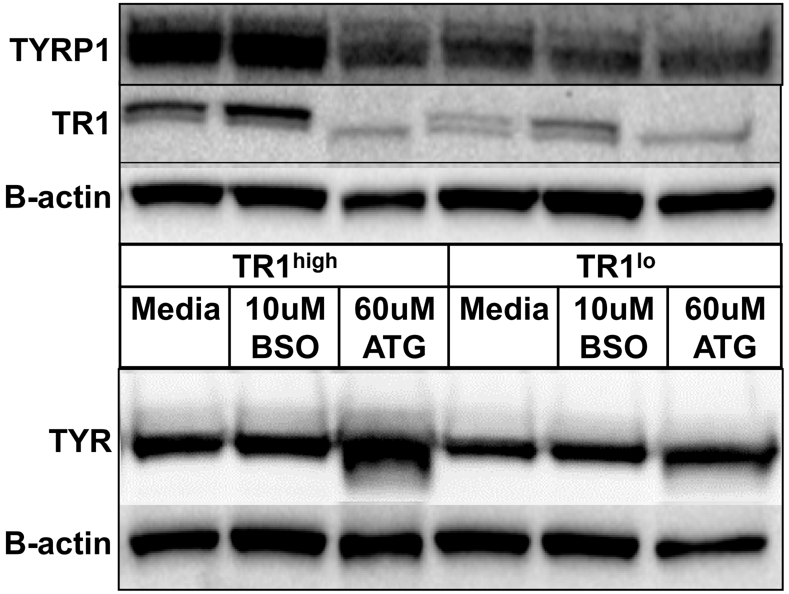

Previous experiment
Serum Lot B

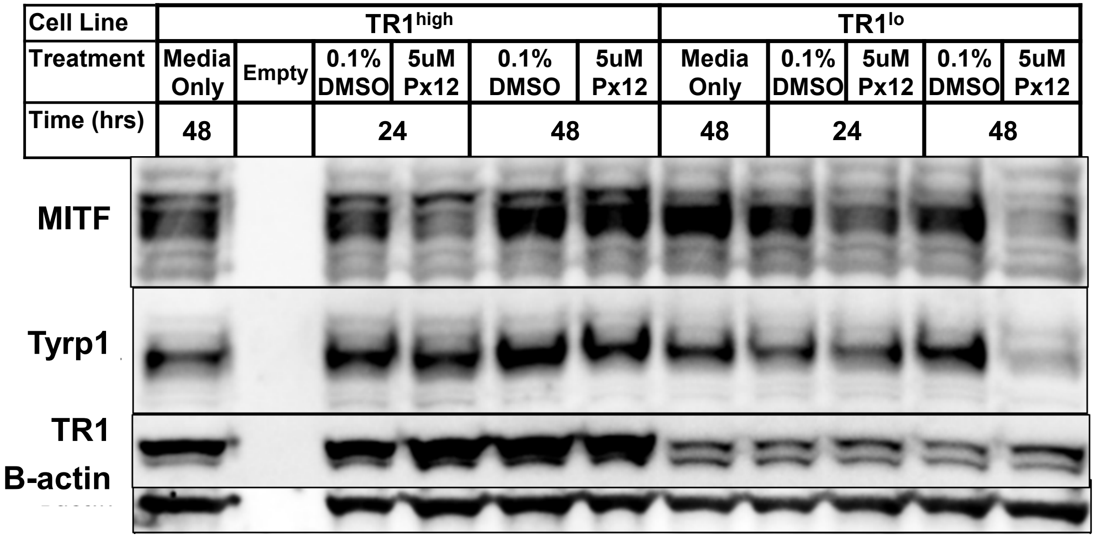

Supplementary Figure S9.

Immunoblot with anti-Trx1

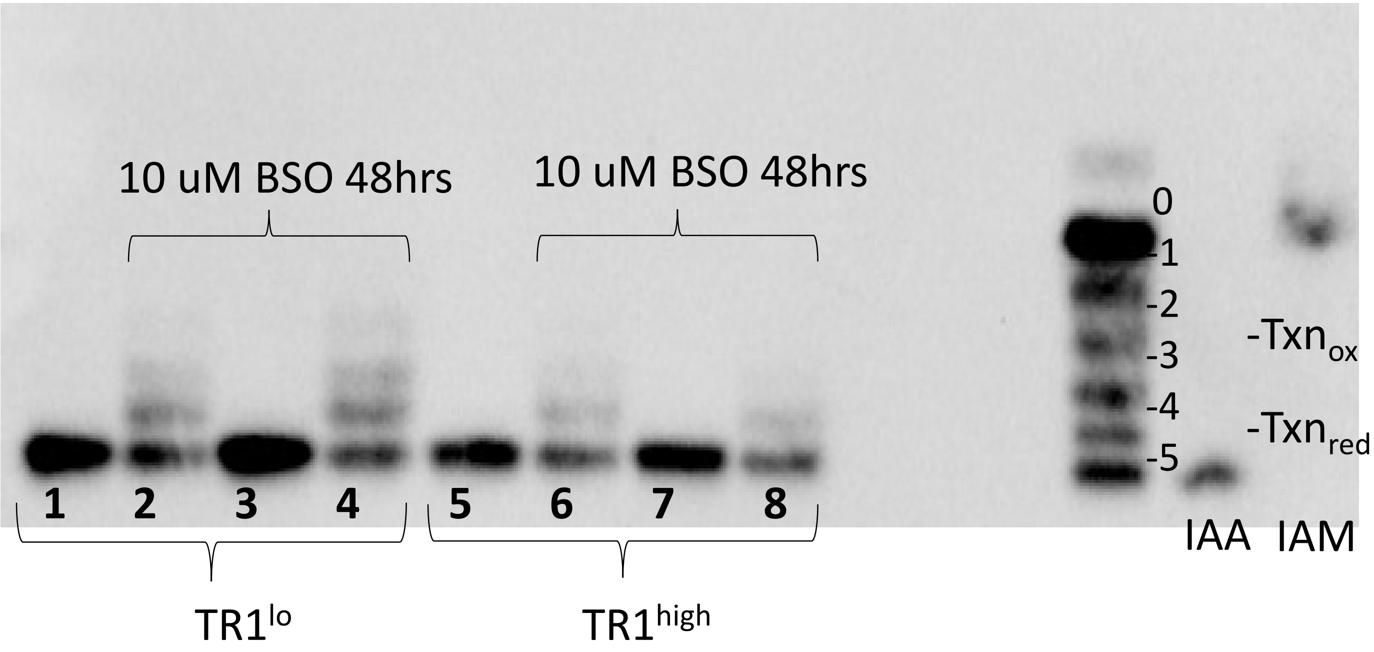

Lanes:
1) IAA
2) IAM
3) BSO + IAA
4) BSO + IAM
5) IAA
6) IAM
7) BSO + IAA
8) BSO + IAM

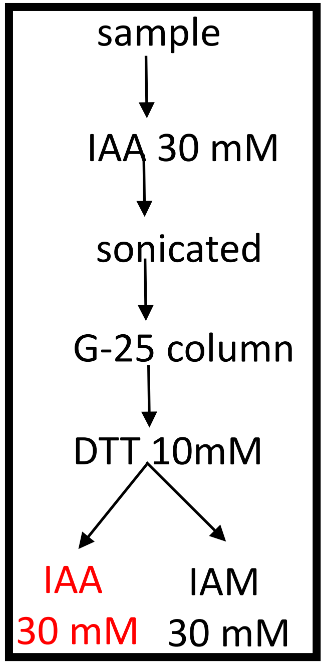

Method:

Fernando M.R., Nanri H., Yoshitake, S., Ngata-Kuno, K., Minakami, S. Eur. J. Biochem. **1992**, *209*, 917-922.

Supplemental Figure S10

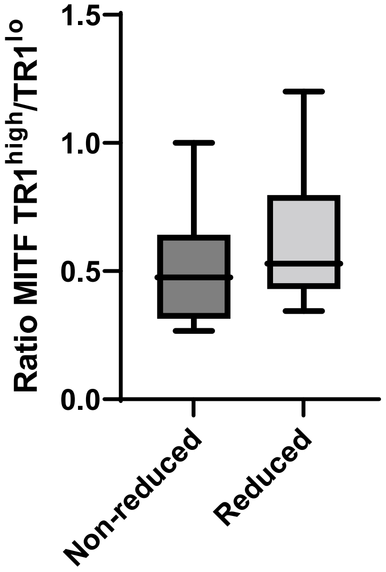

Supplemental Figure S11

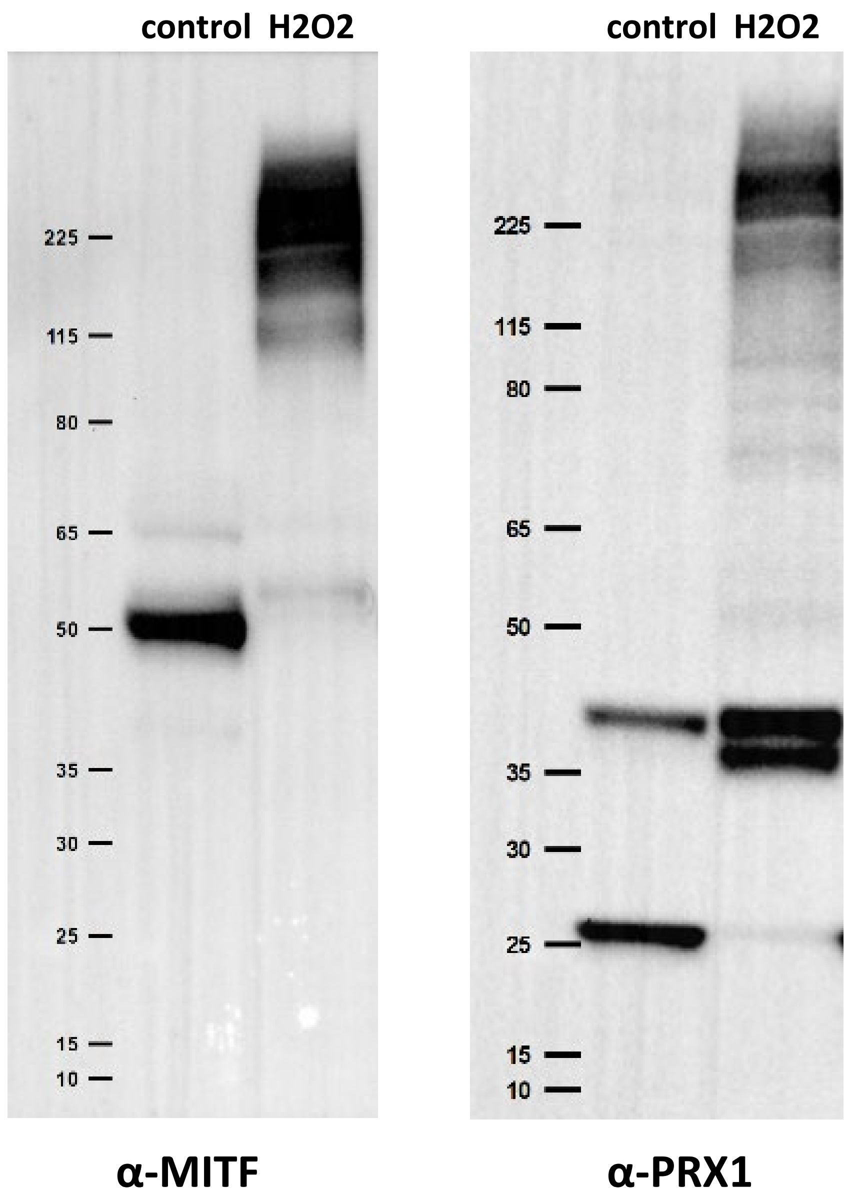

Supplemental Figure S12

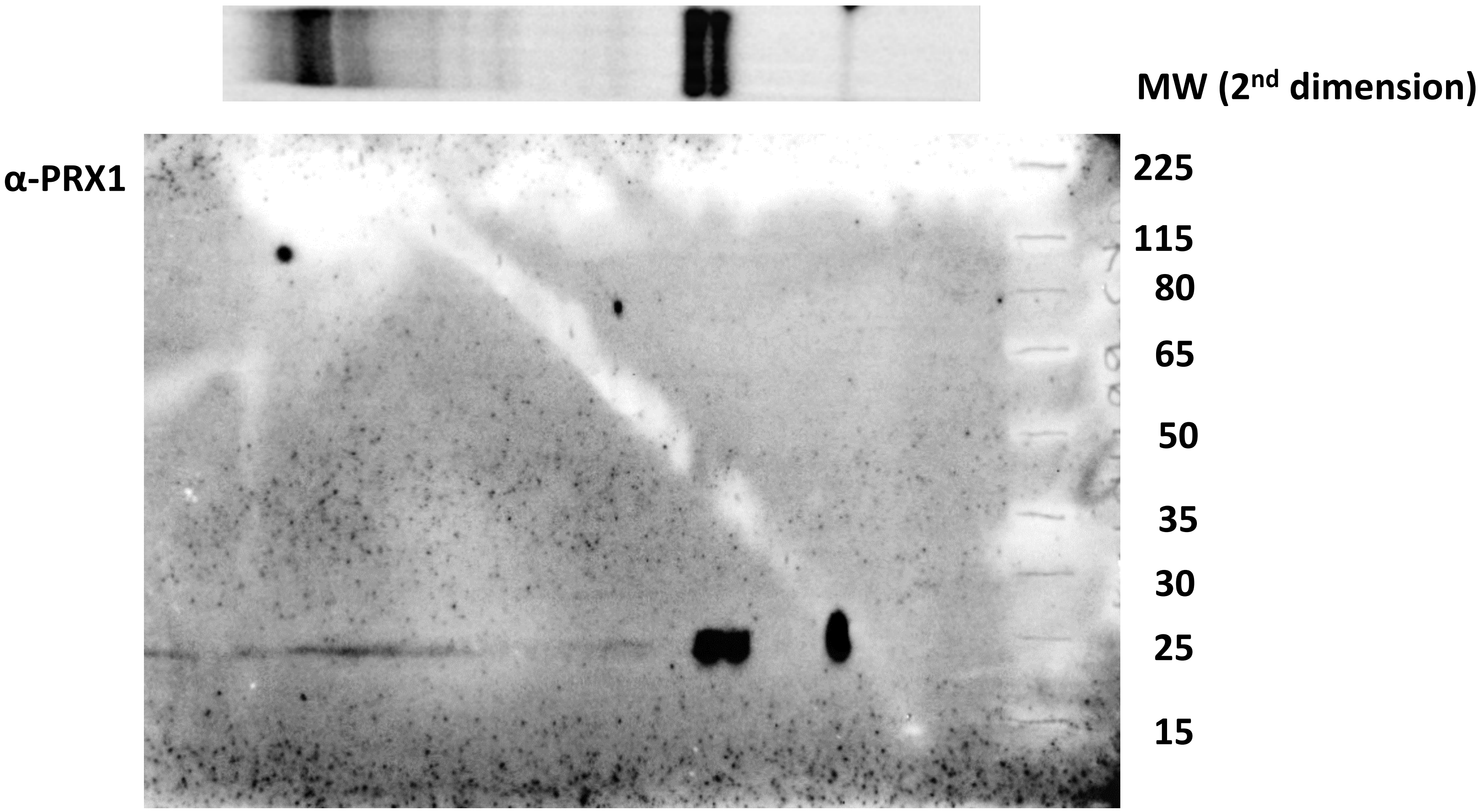

Supplementary Figure S13. Non-reducing

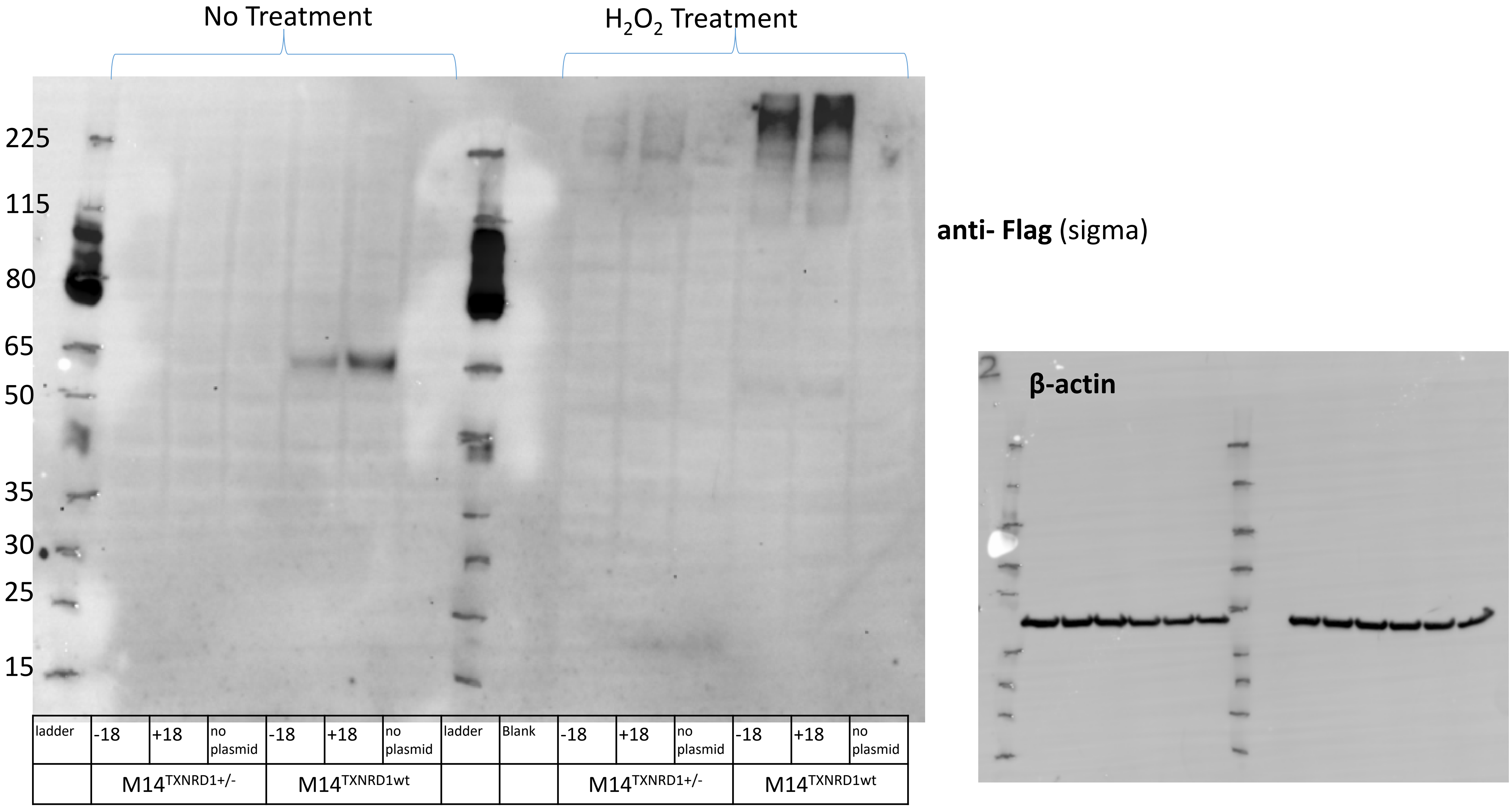

Supplementary Figure S14- Reducing

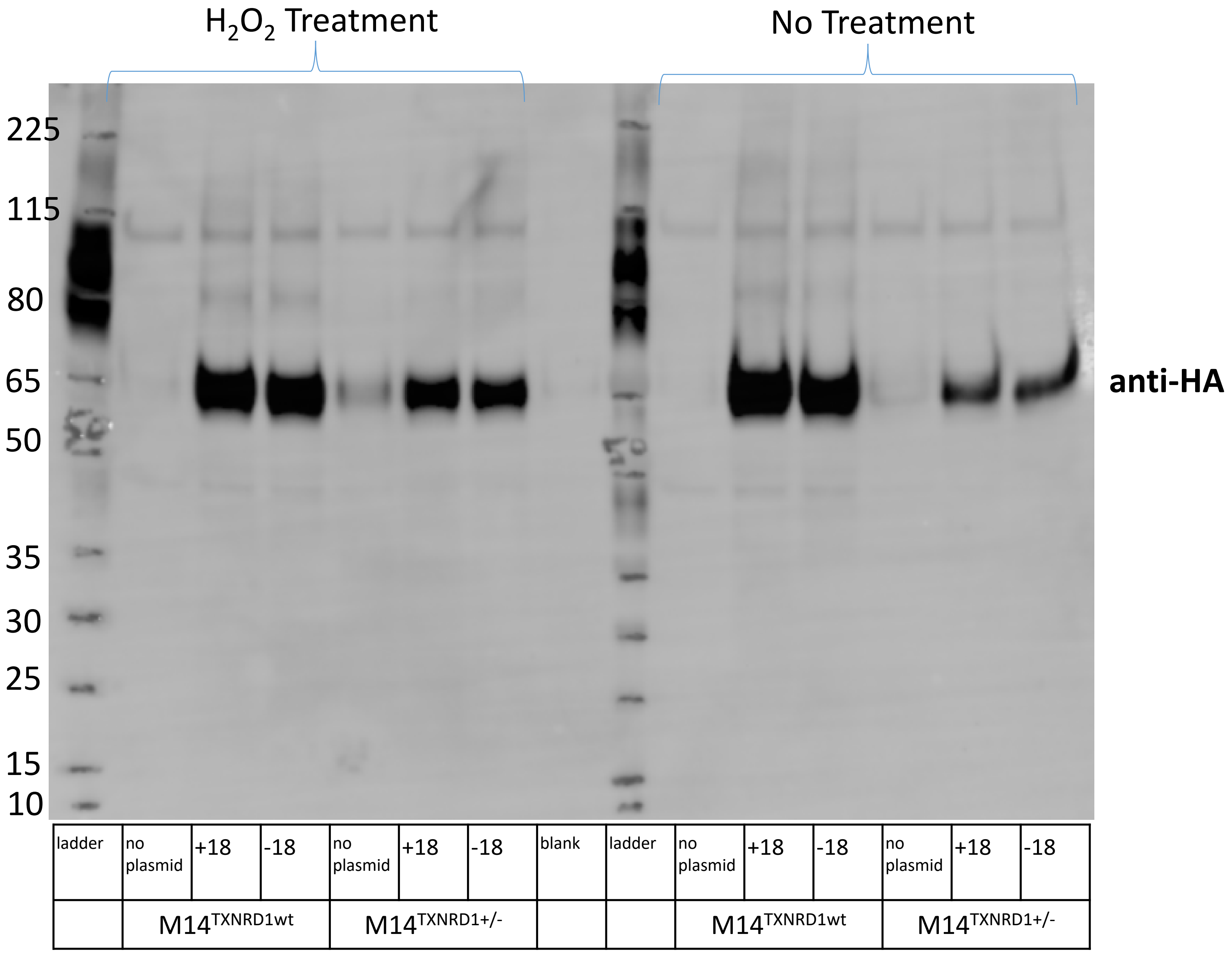

Supplementary Figure S15

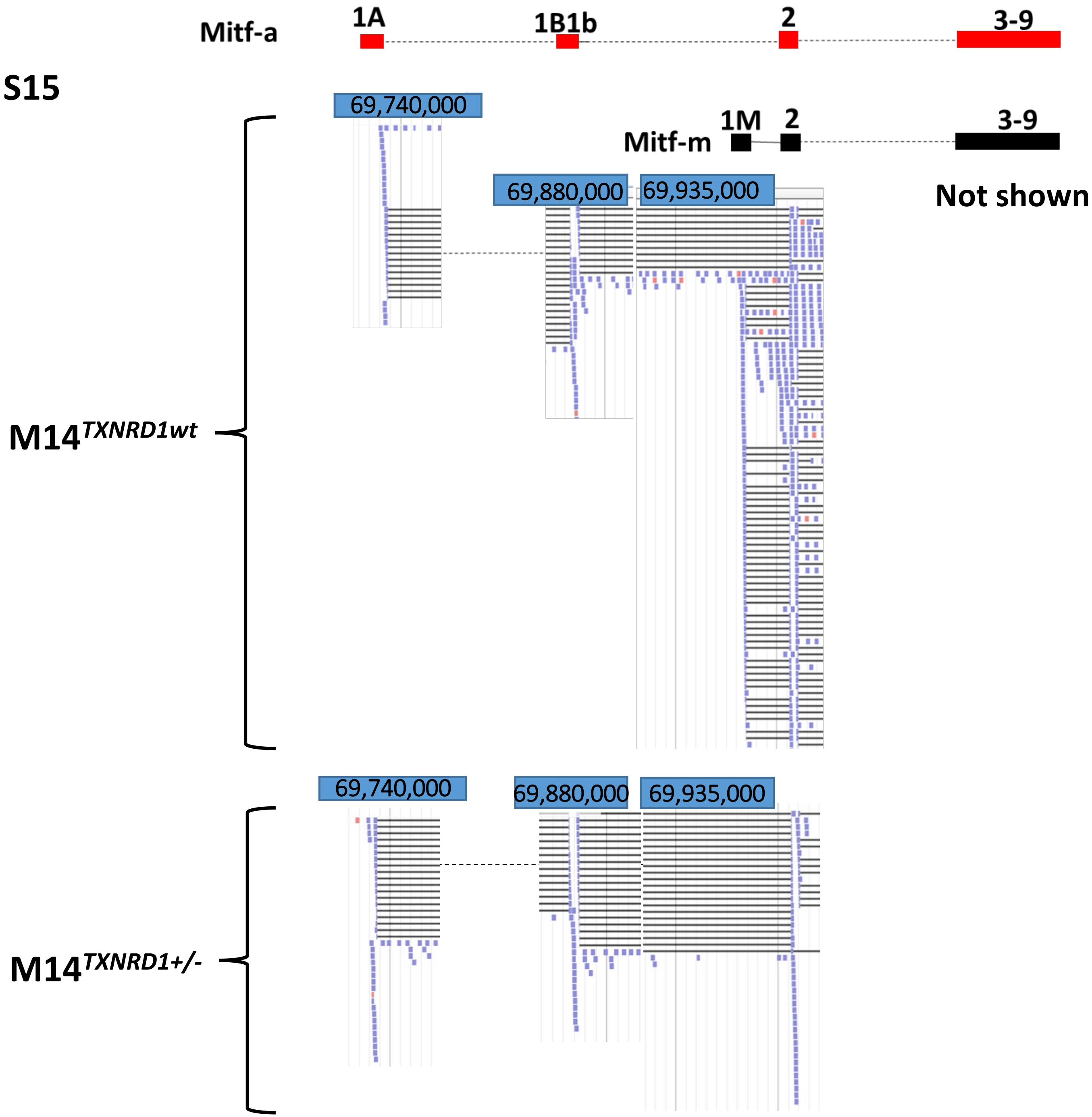

Supplementary Figure S16

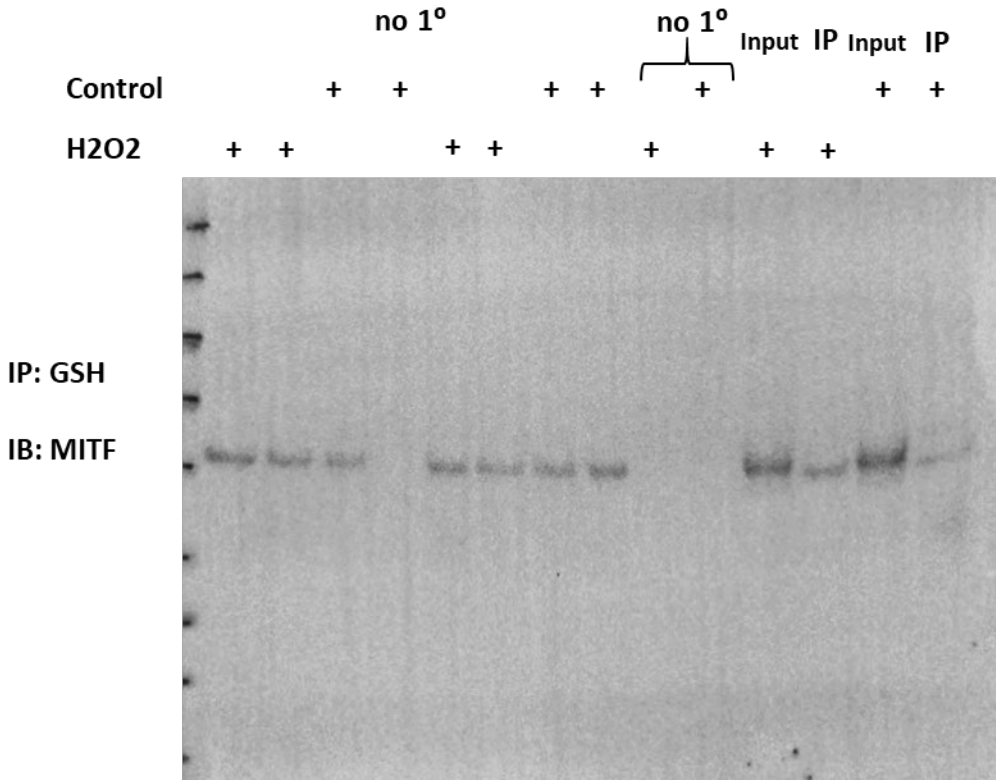

Supplementary Table S1. qPCR Primers

	Forward Primer 5'	Reverse Primer 5'
GCLM	tgg gca cag gta aaa cca a	cag tca aat ctg gtg gca tc
SLC7A11	cag aag ctg cag tta gcc aag	atg aag tct cgc gct ctt gt
SLC1A4	ttt gcg aca gca ttt gct ac	tgt tct ctt caa tgc act tca tc
GSR	ttc aat gat cag cac caa cat gc	taa cct cct tga cct ggg aga
TXN	gta gat gtg gat gac tgt cag ga	cac cca cct ttt gtc cct tc
TXNRD1	ttg gaa tcc acc ctg tct gt	cat cca cac tgg ggc tta ac
GCLC	atg cca tgg gat ttg gaa t	aga tat act gca ggc ttg gaa tg
RPLP0	cct cgt gga agt gac atc gt	atc tgc ttg gag ccc aca tt
MITF*	cat tgt tat gct gga aat gct aga a	ggc ttg ctg tat gtg gta ctt gg
TRPM1*	tgt ggg agt tgt tga gca cag aga	atg cct tga aag acc act cct cca
DCT*	gag tac acc ccg act acg tga	ggc gtc ctg gtc cta ata atg
Tyr*	tag cgg atg cct ctc aaa gc	agg agt ggc tgc ttt tct tca
PMEL*	tct ggg ctg agc att ggg	aga cag tca ctt cca tgg tgt gtg
