# Supplementary material for: MITF Is Regulated by Redox Signals Controlled by the Selenoprotein Thioredoxin Reductase 1
Source: Cancers (Basel). 2022 Oct 13;14(20):5011. doi: 10.3390/cancers14205011 (PMC9600194; doi:10.3390/cancers14205011)
Supplement: Supplementary file 1 [file cancers-14-05011-s001.zip › cancers-1836209-Supplementary Materials and Methods.pdf]

SUPPLEMENTARY MATERIALS AND METHODS

Deletion of TXNRD1 using Crispr/Cas9

M14 cells (NCI, Cat# M14, RRID:CVCL 1395) were electroporated with Cas9 protein (System Biosciences, Palo Alto, CA) and the guide RNA *GAGCATCCTATGTCGCTTTGG* that targets exon 9 of *TXNRD1*. Cells were then single-cell cloned and screened for TR1 by Western blot. In the cell line designated $M14^{TXNRD1-/+}$, both wild-type clones and two different clones with disruption of exons 9 of *TXNRD1* were detected by Sanger sequencing of exon 9 in genomic DNA and cloning of cDNA. These analyses are consistent with two different cell lines with heterozygous deletions of *TXNRD1.* Analysis of the *TXNRD1* locus in the M14 cell line does not suggest any copy number variants in the region of chromosome 3 containing TXNRD1 (https://discover.nci.nih.gov/cellminer/). The deletions detected result in frameshifts and premature stop codons. The control wild-type M14 cells were also single-cell cloned but found to have no disruption of *TXNRD1.* All M14-derived cell lines were analyzed by STR analysis and were found to be identical to the parent cell line.

Protein analysis and western blot without N-ethylmaleimide (NEM) treatment

At the end of experiments, cells were removed by trypsinization, pelleted, and washed with PBS and then lysed in buffer (50 mM Tris pH 7.4, 0.1 M NaCl, 2 mM EDTA, 0.1% SDS, 0.1% deoxycholate) as well as protease and phosphatase inhibitors (Roche). Protein estimations were made by Bradford assay (Pierce) using bovine serum albumin as a standard. Equal amounts of protein lysate were loaded onto 4-12% NuPage Bis-Tris gels (ThermoFisher Scientific) with NuPage 4X LDS sample buffer (ThermoFisher Scientific) and then electrophoresed approximately 90 minutes using a constant voltage of 100V. Proteins from the gels were then transferred to a 0.45 μm PVDF membrane at 30V and 4°C for one hour, and then blocked in 5% non-fat dried milk (NFDM). The membranes were subsequently probed with primary antibodies including Thioredoxin reductase 1 (TR1, Santa Cruz sc-28321, RRID:AB_628405), Tyrosinase (Tyr, Hearing lab PEP7h), Tyrosinase related protein-1 (Tyrp1, Hearing lab PEP1h), Thioredoxin 1 (TRX1, Sigma HPA047478, RRID:AB_2680063), Micropthalmia-associated transcription factor (MITF, Santa Cruz sc-56725, RRID:AB_784547), and β-actin (Cell Signaling, 8457S, RRID:AB_ 10950489) as a loading control. The SuperSignal West Dura Chemiluminescent Substrate detection system was applied before exposing the membrane on the ChemiDoc Touch system for imaging and further analysis (Bio-Rad).

Analysis of H_2O_2-induced protein oxidation (Western blot after treatment with NEM and SDS-PAGE under reducing and non-reducing conditions)

Cells were plated in six-well plates and treated with 1-100 μM H_2O_2 for 1 minute. Cells were processed for analysis following a protocol adapted from Li and Kast [1]. Peroxide solutions were removed and free thiols were blocked by treatment of each well with 100 mM NEM in PBS at 4 C for five minutes. NEM was removed, and cells were lysed on the plate in 100 μL lysis buffer containing 40 mM HEPES pH 7.6, 50 mM NaCl, 1mM EDTA, EDTA-free complete Protease Inhibitor Cocktail tablets (Roche), 20 mM NEM, and 1% peroxide-free Triton X-100. After heating at 37 C for 1 hour, lysates were sonicated. Protein concentration was estimated using the bicinchoninic acid (BCA) method using a kit

purchased from ThermoFisher. Proteins were subjected to SDS-Page and western blotting using non-reducing (NuPage 4X LDS) loading buffer and separately under reducing (1.25% v/v β-mercaptoethanol final concentration) conditions.

RNA Isolation for cDNA synthesis, and Quantitative PCR

For RNA isolation cell pellets were disrupted in 700 μL Qiazol (Qiagen) and total RNA was isolated using the PureLink RNA Mini Kit (Invitrogen) and quantified to ensure 250 ng of each sample was reverse transcribed to cDNA using the SuperScript IV Kit (ThermoFisher Scientific). Samples were prepared for RT-qPCR using the Rotor-Gene SYBR Green PCR Kit (Qiagen) and run on the Rotor-Gene Q (Qiagen) according to the manufacturer's protocol. Reaction mixtures were incubated at 25°C for 10 min, 48°C for 1 h, and 95°C for 5 min. The qPCR reactions were conducted using the primers that were validated and listed in Table 1 at a final concentration of 4 μM. For each reaction, 10 ng of RNA was used with the following conditions: 95°C for 5 minutes, and 39 cycles of 95°C for 10 seconds, 59°C for 20 seconds, and 72°C for 20 seconds. Primers with asterisks, including reference RPLP0, used modified reaction conditions from Khaled et al., 2010 where 20 ng of RNA was subjected to 8.5 minutes at 95°C, and 40 cycles of 95°C for 15 seconds, 62°C for 30 seconds, and 72°C for 20 seconds. At the end of the cycling, a melt curve was generated. The data was analyzed using Rotor-Gene Q Series Software 2.3.1 (Qiagen). Each gene was measured using 4 technical replicates and quantified relative to the housekeeping gene RPLP0 using the ΔΔCT method.

RNA Sequencing

Total RNA was profiled for integrity on a Bioanalyzer (Agilent). The RNA was then incubated with biotinylated ribosomal RNA (rRNA) probes, followed by removal of rRNA using streptavidin-coated magnetic beads. The remaining RNA was fragmented using divalent cations and heat. Fragmented RNA was then converted to cDNA using random hexamer priming. The cDNA second strand was prepared using dUTP in place of dTTP. The ends of the cDNA were blunted, adenylated, and then ligated to standard Illumina sequencing adapters. The ligation products were amplified using PCR with the dUTP in the second strand of the cDNA conferring strandedness. The libraries were profiled on a 2200 TapeStation (Agilent) and quantified using real time PCR (Kapa Biosystems) using a StepOnePlus Real Time Workstation (Thermo/ABI). The libraries were sequenced on a HiSeq 2500 (Illumina) using a single read 100 cycle protocol. Individual base call files were converted to fastq files using Bcl2Fastq (Illumina). The data were aligned to Homo sapiens GR Ch38.95. Differential expression was determined using DESeq2 to normalize the data and limma to evaluate gene expression differences.

Measurement of Glutathione

Reduced glutathione (GSH) was measured using the luminescent-based GSH-Glo Glutathione Assay (Promega). Between 1,500 and 4,000 cells were used per assay in order to avoid interference from melanin. Luminescence was measured on the Synergy H1 microplate reader (BioTek) and expressed in concentrations of nmoles/10^6 cells. To measure the total GSH (reduced plus oxidized GSSG) 1 mM TCEP (Tris(2-carboxyethyl)phospine) was added to the reactions. Oxidized glutathione measurements were calculated by subtracting the total amount of glutathione from the reduced glutathione, and the propagation of error was calculated using the formula below:

$$\hat{\sigma}_{\frac{B}{A-B}} = \frac{\sqrt{A^2\hat{\sigma}_B^2 + B^2\hat{\sigma}_A^2}}{(A-B)^2}$$

where A=amount of total GSH, B =oxidized glutathione, and = the standard deviation of A and B respectively.

Knockdown of TR1 using siRNAs directed against TR1 with three different transfection reagents

M14 melanoma cells were treated with siRNAs targeting human *TXNRD1* (Dharmacon siRNA SMARTpool cat # L-008236-00-0010) or non-targeting control siRNAs (cat# D-001810-01-05) using three different transfection reagents (Dharmacon reagents set of 4 Cat # T-2005-00) according to the manufacturer's instructions. After 48 hours cells were harvested and lysates were analyzed by Western blot.

Immunoprecipitation of glutathionylated proteins and detection of MITF

This protocol was adapted from Poerske et al. [2]. Cells were treated with 100 mM NEM in PBS for 5 minutes at room temperature before lysis on the plates with scraping, in buffer containing 40 mM HEPES pH 7.6, 50 mM NaCl, 1mM EDTA, EDTA-free complete Protease Inhibitor Cocktail tablets (Roche), 20 mM NEM, and 1% peroxide-free Triton X-100. Lysates were sonicated and heated at 37 ^{0}C for one hour, then cleared by centrifugation at 10,000g at room temperature for 15 minutes. Protein concentration in the supernatants was estimated by bicinchoninic acid (BCA) method using a kit purchased from ThermoFisher. Immunoprecipitations were performed using 100 μg protein in total volume of 200 μL. Volumes were adjusted using lysis buffer. One μg of anti-GSH (Virogen Cat#101-A-100) was added to each solution. Controls to which no primary antibody was added were included for each treatment. The mixtures were rotated end-over-end at 4 ^{0}C for 1 hour. Then 20 μL Protein A/G agarose PLUS (Santa Cruz Biotech) was added to each mixture and these were rotated overnight at 4 ^{0}C. The solutions were centrifuged at 250g, 4 ^{0}C for 5 minutes and the supernatant was removed. The beads were washed 3 times with 300 μL lysis buffer with no NEM and with added salt to 150 mM and protease inhibitors. The tubes were rotated at 4 ^{0}C for 5 minutes with each rinse. Protein was removed from beads by heating to 95 ^{0}C in 40 μL 2x loading buffer. The supernatant was then heated with 1 volume of 1M DTT at 95 ^{0}C for 5 minutes before western blot on gels that included samples of input protein prepared under reducing conditions. MITF protein was visualized by probing blots with rabbit monoclonal anti-MITF (Cell Signaling catalog #97800).

References

1. Li, R. and J. Kast; Biotin Switch Assays for Quantitation of Reversible Cysteine Oxidation. *Methods Enzymol*, **2017**. *585*: p. 269-284.
2. Poerschke, R.L., K.S. Fritz, and C.C. Franklin; Methods to detect protein glutathionylation. *Curr Protoc Toxicol*, **2013**. *57*: p. 6 17 1-6 17 18.
